# Perspective on Stem Cell Therapy in Organ Fibrosis: Animal Models and Human Studies

**DOI:** 10.3390/life11101068

**Published:** 2021-10-11

**Authors:** Joanna Wiśniewska, Agnieszka Sadowska, Anna Wójtowicz, Magda Słyszewska, Anna Szóstek-Mioduchowska

**Affiliations:** Institute of Animal Reproduction and Food Research, Polish Academy of Sciences, 10-748 Olsztyn, Poland; j.bukowska@pan.olsztyn.pl (J.W.); a.sadowska@pan.olsztyn.pl (A.S.); a.wojtowicz@pan.olsztyn.pl (A.W.); m.slyszewska@pan.olsztyn.pl (M.S.)

**Keywords:** fibrosis, extracellular matrix (ECM), stem cells, cell-based therapy

## Abstract

Tissue fibrosis is characterized by excessive deposition of extracellular matrix (ECM) components that result from the disruption of regulatory processes responsible for ECM synthesis, deposition, and remodeling. Fibrosis develops in response to a trigger or injury and can occur in nearly all organs of the body. Thus, fibrosis leads to severe pathological conditions that disrupt organ architecture and cause loss of function. It has been estimated that severe fibrotic disorders are responsible for up to one-third of deaths worldwide. Although intensive research on the development of new strategies for fibrosis treatment has been carried out, therapeutic approaches remain limited. Since stem cells, especially mesenchymal stem cells (MSCs), show remarkable self-renewal, differentiation, and immunomodulatory capacity, they have been intensively tested in preclinical studies and clinical trials as a potential tool to slow down the progression of fibrosis and improve the quality of life of patients with fibrotic disorders. In this review, we summarize in vitro studies, preclinical studies performed on animal models of human fibrotic diseases, and recent clinical trials on the efficacy of allogeneic and autologous stem cell applications in severe types of fibrosis that develop in lungs, liver, heart, kidney, uterus, and skin. Although the results of the studies seem to be encouraging, there are many aspects of cell-based therapy, including the cell source, dose, administration route and frequency, timing of delivery, and long-term safety, that remain open areas for future investigation. We also discuss the contemporary status, challenges, and future perspectives of stem cell transplantation for therapeutic options in fibrotic diseases as well as we present recent patents for stem cell-based therapies in organ fibrosis.

## 1. Introduction

**Figure 1:**
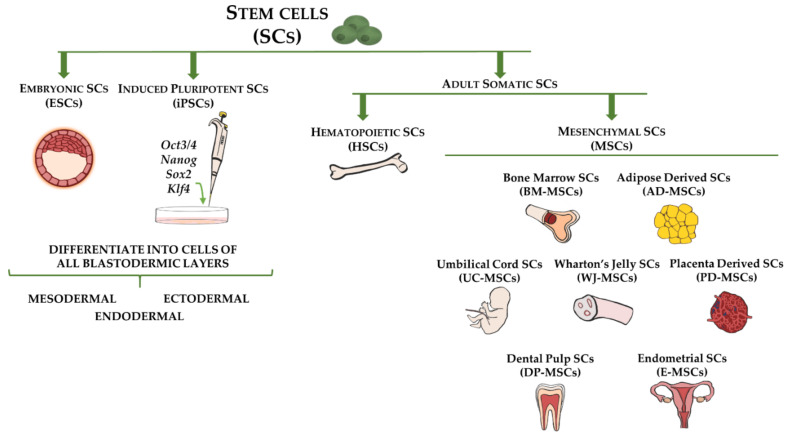
Stem cell types and sources used for cell-based therapy for fibrotic disorders. Embryonic stem cells (ESCs) and induced pluripotent stem cells (iPSCs) are capable of differentiating into cells of all three germ layers (ectoderm, mesoderm, and endoderm). Adult somatic stem cells include hematopoietic stem cells (HSCs), which are multipotent and reside in bone marrow, and mesenchymal stem cells (MSCs), which represent mesodermal progenitors existing in multiple tissues, including bone marrow, adipose tissue, umbilical cord blood, Wharton’s jelly, placenta, dental pulp, and endometrium.

Fibrosis is the excessive accumulation of extracellular matrix (ECM), which leads to impairment of organ function and is consequently associated with high morbidity and mortality. The fibrotic process affects nearly all solid tissues and organs, including the heart, kidney, lung, liver, and skin, and in the majority of cases, this condition results from an ongoing disease (e.g., asthma, hypertension, diabetes, myocardial infarction (MI)) that commonly triggers these tissues [1,2]. Most fibrotic diseases have a relatively well-described etiology, including genetic predisposition, lifestyle, or systemic disorders (Figure 1). Despite showing different clinical features, the majority of fibrotic diseases share common pathological processes characterized by persistent inflammation leading to the production of growth factors, cytokines, and proteolytic enzymes. Altogether, these factors affect myofibroblast differentiation and stimulate excessive deposition of connective tissue components [3].

It has been estimated that severe fibrotic disorders are responsible for up to one-third of deaths worldwide [1]. Researchers and clinicians all over the world have worked on developing efficient therapeutic strategies to treat these life-threatening diseases. However, despite constant efforts, there are still no effective therapies for the treatment of fibrosis in any organ (Figure 1). Stem cell-based therapy shows great promise for restoring injured tissues and treating/reversing fibrotic changes. The vast therapeutic potential of this type of therapy in the treatment of neurodegenerative, autoimmune, and genetic disorders has been reported [4,5]. Moreover, clinically relevant studies have included the application of mesenchymal stem cells (MSCs) in a broad range of degenerative, post-injury, and fibrosis-related diseases [6,7,8].

Mesenchymal stem cells are present in various tissues and organs, including bone marrow, adipose tissue, umbilical cord, and endometrium [9,10,11,12]. According to the standard definition, MSCs are fibroblast-like, adherent, clonogenic cells that express a number of surface markers, such as CD29, CD73, CD90, and CD105, and exhibit the capacity to differentiate into adipogenic, chondrogenic, and osteogenic lineages in vitro [13]. The mechanisms through which transplanted MSCs show their therapeutic effects rely on cell differentiation into tissue-specific cell types that replace defective cells or on the secretion of growth factors that lead to an enhanced renewal of target tissue [14,15]. Notably, in vivo studies using rodent models of degenerative neurological diseases have shown that MSCs might contribute to tissue repair by acting as immunomodulatory, neuroprotective, and anti-inflammatory agents [16,17].

In this review, we summarize the results of recent studies regarding the use of stem cells in experimentally induced and clinical cases of fibrosis in multiple organs as well as we present recent patents for stem cell-based therapies in organ fibrosis. We also discuss challenges, limitations, and future perspectives of cell-based therapies that should be considered before conducting large-scale trials.

## 2. Cellular and Molecular Basis of Tissue Fibrosis

It has been long established that myofibroblasts are the cells responsible for tissue fibrosis [18,19]. Myofibroblasts are heterogeneous cell populations that are defined by the expression of the contractile protein α-smooth muscle actin (αSMA or ACTA2) and the ability to synthesize and release ECM proteins such as collagen (COL) and fibronectin (FN) [18]. A variety of cell types, including resident fibroblasts, vascular pericytes, and bone marrow-derived cells, can be precursors of myofibroblasts [18]. Moreover, in the skin and the lung, epithelial cells can also contribute to the myofibroblast pool as they undergo the epithelial-to-mesenchymal transition (EMT) process [20,21]. Furthermore, as demonstrated in a study by Marangoni et al. [22], subcutaneous adipocytes can transdifferentiate into myofibroblast-like cells upon transforming growth factor beta (TGF)-β stimulation.

The development of fibrotic diseases is associated with abnormal accumulation of myofibroblasts. This aberration leads to excessive deposition of ECM components, which impairs organ structure and function. In healthy tissues, there is a balance between the synthesis and degradation of collagen and other ECM components. These processes are disrupted in fibrotic organs and directed toward ECM accumulation, which is partially evoked by an increased expression of tissue inhibitors of matrix metalloproteinases (TIMPs) versus a decrease in matrix metalloproteinases (MMPs) [23,24]. Indeed, numerous fibrotic-related human liver diseases, including biliary atresia, primary biliary cirrhosis, and primary sclerosing cholangitis, are associated with TIMP-1 and TIMP-2 overexpression [25]. Moreover, membrane-bound matrix metalloproteinase (MT1-MMP)-deficient mice that show a deficiency of membrane-type MMP-14, the metalloproteinase responsible for pericellular proteolysis of ECM, develop progressive fibrosis of the dermis and hair follicles [26].

Although multiple growth factors and pathways contribute to myofibroblast differentiation and EMT process, TGFβ signaling is the major inducer of fibrotic lesions, as has been evident in several in vitro and in vivo studies [27,28]. The activation of TGFβ signaling by overexpression of constitutively active TGFβ receptor type 1 (TGFβRI) is sufficient to induce a fibrotic phenotype characteristic of systemic sclerosis (SS) in mice [29]. In contrast, inhibition of the TGFβ pathway by phosphodiesterase (PDE) inhibitors exert potent antifibrotic effects in human lung fibroblasts [30]. TGFβ signaling is transduced through Smad and non-Smad pathways, such as mitogen-activated protein kinase pathways mediated by extracellular-signal-regulated kinase (ERK), RHO-associated kinase (ROCK), p38 and JUN N-terminal kinase (JNK), and RAC-α serine/threonine-protein kinase pathways [31]. Moreover, TGFβ1 stimulates the secretion of secondary signals, such as connective tissue growth factor (CTGF), plasminogen activator inhibitor 1 (PAI-1), endothelin-1, and NADPH oxidase 4 [32]. These agents show the capacity to mediate and/or mimic the effects of TGFβ1 and ultimately participate in signaling crosstalk, which consequently results in abnormal tissue remodeling and fibrosis in several organs [32].

At present, there is a large amount of data indicating that Wnt and hedgehog signaling, well-described developmental (morphogen) pathways, are potent modulators of fibrotic tissue remodeling across organs [33,34]. Overexpression of Wnt1 and Wnt10b proteins has been observed in human samples from SS, idiopathic pulmonary fibrosis (IPF), and liver cirrhosis [33]. Moreover, it has been shown that β-catenin, the central component of the canonical Wnt signaling, accumulates in the nuclei of fibroblasts from patients with SS and is overexpressed in the skin of mice with bleomycin (BLM)-induced SS. Interestingly, in parallel with the upregulation of Wnt ligands, a significant decrease in the endogenous Wnt antagonist Dickkopf-1 (Dkk-1) was reported [33]. Similarly, other studies demonstrated increased expression of sonic hedgehog (SHH) and the hedgehog transcription factor GLI2 in the skin of patients with SS [35]. At the same time, in the serum of those patients, high SHH concentrations were observed, correlating with the fibrotic burden [36]. Besides the aforementioned key regulators of fibrosis, there is a wide range of well-established factors that stimulate fibrogenesis, such as inflammatory mediators and cells, hypoxia, and DNA methylation of antifibrotic genes [37,38,39]. Importantly, it must be highlighted that tissue fibrosis is a complex process that involves the action of many factors and the activation of multiple signaling cascades that cross-react at multiple levels, and consequently, this condition presents a challenge in the development of novel antifibrotic approaches.

## 3. Stem Cell Types for Fibrotic Disorders Therapy

Although stem cells exist in relatively low quantities in adult tissues, they comprise a unique cell population that greatly contributes to tissue homeostasis and drives tissue regeneration [40]. By definition, stem cells are characterized by their ability to self-renew and differentiate into multiple cell lineages, which could provide therapeutic solutions for numerous diseases [6,41]. Although multiple criteria are used to classify stem cells (e.g., origin, differentiation potency), generally they can be categorized as embryonic stem cells (ESCs), collected from pre-implantation blastocysts, induced pluripotent stem cells (iPSCs), representing genetically reprogrammed adult somatic stem cells to an ESC-like state, and postnatal adult somatic stem cells, obtained from adult tissues (Figure 1) [42].

Embryonic stem cells are pluripotent cells that possess unlimited developmental potential, reflected by their capacity to form all types of tissues in the body. This ability to differentiate into multiple mature somatic cell types is maintained in cell cultures. The pluripotency fate of ESCs is driven by transcription factors Oct4, Sox2, and Nanog, which are termed pluripotency factors. However, upon appropriate stimulation, ESCs initially form 3D spherical structures termed embryoid bodies (EBs), then differentiate into precursor cells, and further give rise to various somatic cell lineages [43]. Indeed, the in vitro capacity of mouse ESCs to produce functional cardiomyocytes, chondrocytes, osteoblasts, endothelial cells, alveolar epithelium, and neuroectodermal cells, among others, has been demonstrated [44,45,46,47,48]. At present, ESC lines are available from the embryos of numerous mammalian species, including humans [49]. Although the generation of human ESC lines and their use in scientific research and clinical practice has raised an ethical controversy, it is important to acknowledge that differentiated cell lineages from ESCs have had an enormous impact in many fields of research, and they can serve as a promising tool for regenerative medicine in the treatment of a wide range of degenerative disorders.

The development of methods for reprogramming somatic cells into a pluripotent-like state and generating iPSCs via genomic integration and high expression of four Yamanaka factors (Oct4, Sox2, Klf4, and c-Myc) allowed researchers to overcome the obstacles related to the use of ESCs [50]. Even more important, since iPSCs share the same regenerative properties as ESCs, they hold great promise as custom-made pluripotent cells that could be produced for individual patients and further used in autologous transplantation. One of the advantages of iPSCs in clinical application is the fact that the cells are unlikely to cause host immune rejection [51]. Recent in vitro studies demonstrated the ability of iPSCs to differentiate into beating cardiomyocyte-like cells, insulin-producing islet-like clusters (ILCs), and neuronal and glial cell types [52,53,54]. Of particular interest, several iPSC-based clinical trials, mostly targeting eye diseases, are under way, highlighting the enormous progress that has been made in stem cell biology and regenerative medicine [55].

The most common adult somatic stem cells, which have been used for the longest time, are MSCs. They represent mesodermal progenitors existing in multiple tissues, including bone marrow, adipose tissue, umbilical cord blood, dental pulp, and endometrium (Figure 1) [11,12,56,57,58]. The use of MSCs allows researchers to overcome the ethical and legal issues associated with the application of ESCs and the uncontrolled mutational effects related to iPSCs. Although bone marrow was the first organ studied as a source of MSCs (BM-MSCs), cells isolated from other tissues, including adipose tissue, periodontal ligament, and trabecular bone, demonstrated comparable characteristics in terms of morphology, surface markers, and differentiation potential in vitro [59]. Moreover, limitations related to BM-MSC procurement, such as the high risk of morbidity associated with the bone marrow aspiration procedure and the relatively low yield of isolated MSCs (0.001–0.01% of harvested bone marrow cells), have led to a search for alternative sources of MSCs [59,60]. In this regard, adipose-derived stem cells (ASCs) overcome the obstacles of BM-MSCs, as these cells can be isolated from multiple fat depots in large quantities using a minimally invasive procedure, such as liposuction or other surgical interventions that in humans are used for the removal of excess fat [61]. Several studies have demonstrated the plasticity of human and animal ASCs [62,63,64]. Consistently, there is extensive literature regarding the safety and efficacy of ASC-based therapies for the treatment of numerous diseases and injuries created in animal models of human diseases, including cardiovascular disease and musculoskeletal and cutaneous injuries [65]. The therapeutic potential of ASCs or Stromal Vascular Fraction cells (SVFs) has been confirmed not only in experimental animal models but also in an increasing number of human clinical trials [66,67,68].

### 3.1. Pulmonary Fibrosis

**Figure 2:**
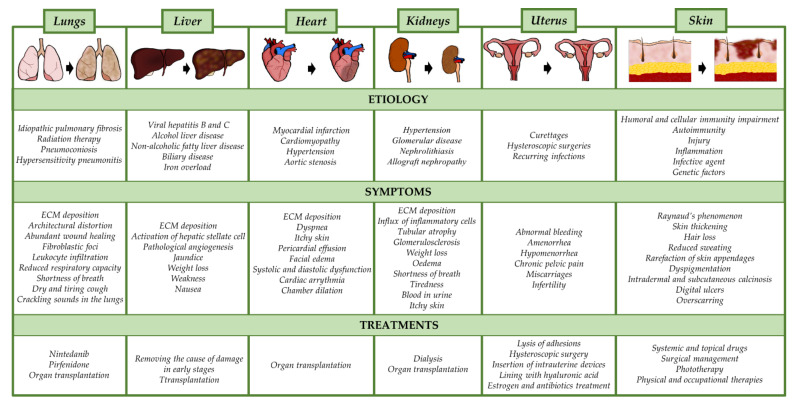
Etiology, symptoms, and current treatment options for fibrotic diseases in individual body organs. ECM; extracellular matrix.

Pulmonary fibrosis (PF) is associated with over 200 chronic lung diseases, which differ in the severity of their course, the degree of inflammation, and the advancement of fibrotic foci. The most common and lethal form of idiopathic interstitial pneumonia is idiopathic pulmonary fibrosis, which affects 3–9 per 100,000 people annually, with poor median survival rates at 2–3 years and 5-year survival ranging between 30% and 50% [69]. Many PF disorders emerge from underlying causes such as autoimmune diseases, including SS and rheumatoid arthritis. The important factors that contribute to the development of PF include smoking, hazardous chemicals, air pollution, exposure to cancer radiation therapy or chemotherapy, and genetic and epigenetic factors (Figure 2).

So far, only pirfenidone and nintedanib have been approved as pharmacological therapies for the treatment of IPF (Figure 2). These drugs used clinically are effective in prolonging the time of disease progression, slowing down the decline in lung function, and improving the quality of life of patients with IPF [70,71]. However, damaged lung tissue is not restored with these drugs, and both drugs are associated with gastrointestinal adverse events. Hence, there is an urgent need to establish novel treatment strategies for lung fibrosis, such as stem cell therapy.

Several in vivo studies using animal models of human PF demonstrated promising effects of using stem cells [72,73]. Indeed, a study by Ortiz et al. [72] showed that systemic administration of 5 × 10^5^ BM-MSCs in mice with BLM-induced lung fibrosis protects the lungs from injury by reducing inflammation, collagen deposition, and MMP (MMP-2, -9, -13) activation in lung tissue. More interestingly, MSCs have been shown to engraft in lung tissue, where they acquire epithelial-like morphology, indicating that they might exert their effect not only via regulation of the local environment but differentiation and structural support of injured tissue [72]. These findings were further supported in a study by Rojas et al. [73], which demonstrated the presence of green fluorescent protein (GFP)-positive BM-MSCs in lungs 14 days after BLM treatment and subsequent cell transplantation. Furthermore, following BM-MSC delivery, the authors observed an increase in circulating levels of granulocyte colony stimulating factor (G-CSF) and granulocyte-macrophage colony-stimulating factor (GM-CSF), which may be responsible for mobilizing stem cells from bone marrow pools, suggesting that the therapeutic effect of BM-MSCs might rely, at least in part, on the mobilization of endogenous stem cells. The action of BM-MSCs was also manifested by the suppression of prolonged BLM-caused inflammatory response in the lungs, as this effect was shown by a decrease in mRNA expression of interferon (*IFN*)*-γ*, and *interleukin* (*IL*)-*2*, *IL-1β*, and *IL-4* in lung tissue at 14 days after exposure to BLM and stem cell delivery [73].

Many investigators have used allogeneic mouse or human ASCs (hASCs) as possible treatments for BLM-induced lung fibrosis in mice [74,75]. In one such study, hASCs pooled from five donors were administrated intravenously at a dose of 4 × 10^7^ cells/kg body weight on days 3, 6, and 9 post-BLM administration [75]. hASC administration resulted in significant inhibition of BLM-induced lung fibrosis, reflected by a reduction in collagen deposition and downregulation of the mRNA of proinflammatory cytokines (*IL-2*, *IL-1β*, *tumor necrosis factor (TNF)*, *TGFβ1*), leading to a drop in mRNA expression of factors responsible for ECM deposition and remodeling, such as *basic fibroblast growth factor* (*bFGF*); *Timp-1*, *-2*, and -*3*; *Mmp-2* and *-3*; *CTGF*; and *Col1a1* and *Col1a3* in lung tissue. Notably, histological analysis of lungs confirmed by high-resolution computed tomography exhibited attenuation of lung fibrosis accompanied by thickening of alveolar septa and maintenance of alveolar architecture [75]. Similarly, Kotani et al. [74] demonstrated inhibition of pulmonary inflammation and fibrosis upon intravenous infusion of mouse ASCs (mASCs) at a dose of 2.5 × 10^4^ or 2.5 × 10^5^ in mice with BLM-induced lung fibrosis. The anti-inflammatory effect of mASCs was exhibited by reduced numbers of infiltrated macrophages, neutrophils, and T lymphocytes in lung tissue as compared to control (BLM injected but not mASC-treated animals). Moreover, an in vitro study carried out on macrophages co-cultured with mASCs revealed significant downregulation of the mRNA of proinflammatory cytokines, *TNF-α* and *IL-12* in activated macrophages and increased macrophage apoptosis. Additionally, the results of this study indicated that mASCs inhibited the differentiation and proliferation of Th2 cells, suggesting their role in promoting the differentiation and proliferation of T-regs, which might provide the mechanism based on which mASCs reduce pulmonary inflammation [74].

Important results indicating that the therapeutic efficacy of ASCs may vary with age were obtained by Tashiro et al. [76], who used 5 × 10^5^ mASCs from young (4 months) and old (22 months) male mice as a treatment for BLM-induced lung fibrosis in an aged mouse model (>22 months). Decreased fibrosis, evidenced by reduced interstitial and perivascular collagen deposition estimated histologically and based on hydroxyproline content, was found in aged mice 21 days after transplantation of mASCs obtained from young animals. Moreover, delivery of mASCs from young donor mice led to multiple changes in fibrotic lungs, as manifested by a drop in mRNA expression of *TGFβ*, inhibition of Mmp-2, and protein kinase B (Akt activity, maintenance of redox balance, and reduced cell apoptosis. In contrast, delivery of old-donor ASCs into aged BLM-treated mice did not exert such remarkable effects, as they did not reduce fibrosis and its related markers. Undoubtedly, this study indicates that the antifibrotic properties of ASCs are age-dependent, and this should be considered in clinical trials in patients with PF [76].

There have also been studies investigating the use of umbilical cord MSCs (UC-MSCs) derived from Wharton’s jelly for the treatment of lung fibrosis that support the antifibrotic and immunomodulatory properties of stem cells [77,78]. As reported by Moodley et al. [78], systemically administered human UC-MSCs (hUC-MSCs; 1 × 10^6^) in mice with BLM-induced lung fibrosis reduced levels of inflammatory and profibrotic markers at 14 days. Indeed, a decrease in mRNA expression of IL-10, TNF-α, TGFβ, and IFN-γ in lung tissue was shown following UC-MSC treatment. Furthermore, the cell delivery attenuated collagen deposition and improved the balance between MMP-2 and TIMP 1–4 in lung tissue that was abrogated upon BLM treatment [78]. Interestingly, others showed that intraperitoneal or intratracheal application of fetal membrane-derived MSCs from both humans and mice led to decreased neutrophil infiltration and a significant reduction in the severity of BLM-induced lung fibrosis regardless of the cell source (allogeneic or xenogeneic) or delivery route [77].

Preclinical results showed that iPSCs might provide new therapeutic opportunities in damaged lungs [79]. In a mouse model of BLM-induced lung fibrosis, Zhou et al. [79] demonstrated a significant therapeutic effect mediated by alveolar epithelial cells (AECs) differentiated from iPSCs. After 12 days, it was shown that the cells integrated into the lung alveolar structure and expressed lung progenitor markers such as surfactant protein C and T1α. Importantly, differentiated iPSCs contributed to the reconstitution of BLM-damaged lung tissue by diminishing inflammation and fibrosis [79]. Similar results have been shown using iPSCs generated from mouse embryonic fibroblasts and delivered at a dose of 2 × 10^6^ in a mouse model of BLM-induced lung fibrosis. In that study, inhibition of collagen accumulation in the lungs decreased infiltration of inflammatory cells, and downregulation of TNF-α, IL-1β, and IL-6 at 21 days was reported. Moreover, iPSC transplantation blocked TGFβ1/Smad2/3 signaling, which is a key profibrotic pathway and potential therapeutic target in lung fibrosis [80]. In another study that focused on iPSC secretome, the culture medium containing hepatocyte growth factor (HGF) secreted by iPSCs contributed to AECs repair in vitro and attenuated BLM-induced lung fibrosis in vivo [81].

Many successful preclinical studies in rodents have encouraged the translation of stem cell-based therapy into clinical settings [82,83,84,85]. Currently, multiple clinical trials are being conducted to ensure the safety of stem cell treatment for IPF (https://ClinicalTrials.gov; accessed 16 September 2021) (Table 1). A non-randomized, non-placebo-controlled phase I trial named idiopathic pulmonary fibrosis via intravenous delivery (AETHER) showed the safety and efficacy of intravenous delivery of allogeneic BM-MSCs. At 36 weeks follow-up, improvement in lung function was found based on the results of 6-min walk test (6MWT), and at 60 weeks post transplantation, a decrease in the predicted value of forced vital capacity (FVC) by 3.0% on average and a 5.4% mean decline in carbon monoxide diffusing capacity (DLCO) were observed [84]. Another study carried out on IPF patients who received 1 × 10^7^ or 1 × 10^8^ of allogeneic BM-MSCs demonstrated slower progression of lung fibrosis, especially with the higher cell dose [83]. Averyanov et al. [82] conducted a phase I study in which allogeneic BM-MSCs were administrated to IPF patients in a high cumulative dose of 2 × 10^8^ cells every 3 months (1.6 × 10^9^ cells total). Patients were followed for 52 weeks, and no significant adverse effects were reported. In the group of MSC recipients, lung function increased compared to the placebo group. Indeed, improvements in 6MWT at 13 weeks, DLCO at 26 weeks, and FVC at 39 weeks were observed compared with placebo. Significantly, in the MSC therapy group, at 12 months, FVC increased by 7.8% from baseline, whereas it declined by 5.9% in the placebo group [82]. Tzouvelekis et al. [85] conducted a non-randomized, no placebo-controlled, phase Ib clinical trial in which IPF patients received autologous SVFs in three endobronchial infusions at a dose of 0.5 × 10^6^ cells/kg body weight. They found at 12 months follow-up that 86% of patients had a stable function and exercise capacity. Safety monitoring indicated that cell-administered patients did not deteriorate in either functional parameters or indicators of quality of life [85].

Although two new drugs, nintedanib, and pirfenidone, have been found to be clinically effective in reducing the progression of PF and improving patients’ quality of life, the damaged lung tissue does not recover with these drugs. Therefore, taking into consideration the advantages of using stem cell therapy, their use in the treatment of PF seems to be highly desired. Notably, the potential effects of MSCs in PF mostly rely on their ability to secrete biologically active agents that show immunosuppressive, anti-inflammatory, and pro-angiogenic properties.

### 3.2. Liver Fibrosis

Liver fibrosis, a condition that frequently progresses to cirrhosis, is one of the major challenges of global health. It is estimated that nearly 7% of the population suffers from liver fibrosis, and every year more than 2 million people die worldwide due to fibrosis-associated liver failure [86]. The main causes of hepatic fibrosis include non-alcoholic steatohepatitis (NASH), non-alcoholic fatty liver disease (NAFLD), alcohol abuse, hepatitis B (HBV) and hepatitis C (HCV) virus infections, autoimmune hepatitis, iron overload, and biliary obstruction (Figure 2) [86]. While the early stages of hepatic damage are reversible, the severe stages of fibrotic diseases, especially advanced cirrhosis, are not, and they lead to subsequent morbidity and mortality [87].

Healthy liver tissue consists of around 3% ECM in the area of interest in a normal cross-section, mainly located around Glisson’s capsule, portal tracts, sinusoid walls, and central veins [88]. As fibrosis develops, the ECM content increases up to five-fold. Interestingly, the liver has a unique ability to slow down or even reverse fibrosis, and after removal of profibrotic factors, activated hepatic stellate cells undergo apoptosis, while MMPs break down excessive ECM. However, if the exposure to damaging factors is permanent, hepatic stellate cells migrate and proliferate drastically, altering their phenotype and secreting large amounts of ECM [89].

A study on a mouse model of liver fibrosis induced by carbon tetrachloride (CCl_4_) infusion demonstrated that administration of 1 × 10^6^ BM-MSCs decreased liver fibrosis at 4 weeks follow-up [90]. Histological analysis of the liver demonstrated collagen reduction and recovery of tissue architecture as a consequence of BM-MSC administration. The mRNA expression of *αSMA* and *Timp-1* was downregulated following BM-MSC treatment. Moreover, there were some engrafted cells in the recipient liver that were able to differentiate into albumin-positive cells [90]. These results are in agreement with studies performed on rats with CCl_4_-induced liver fibrosis that received BM-MSCs at a dose of 3 × 10^6^ [91]. In the cell-treated group, a significant decrease in collagen deposition and a decrease in hydroxyproline content were evident in the liver. An elevation in serum albumin (ALB) and a decrease in alanine aminotransferase (ALT) levels were also detected following cell transplantation [91]. Likewise, Zhao et al. [92], using a rat model of CCl_4_ or dimethylnitrosamine-induced liver injury, demonstrated a reduced mortality rate mediated by BM-MSCs delivered at a dose of 3 × 10^6^ cells. Consistent with other studies, decreased deposited collagen content and weaker αSMA staining in the liver of MSC-treated rats was found [92].

An important study underlining the source-specific potential of stem cells to exert a therapeutic effect was conducted using allogeneic MSCs, hematopoietic stem cells (HSCs), or combined MSCs + HSCs (1 × 10^6^ cells) as a treatment for induced liver injury [93]. A comparable analysis revealed that MSCs demonstrated more efficient reparative activity than HSCs with no synergistic effects between the two types of cells. After transplantation of MSCs, the injured livers exhibited maximal restoration with thinner fibrotic areas and decreased collagen levels relative to the HSC- or MSC + HSC-treated groups. Mice receiving MSCs showed superior improvement of liver function, as demonstrated by decreased serum albumin (ALB), ALT, and aspartate aminotransferase (AST) levels in the peripheral blood. Moreover, in animals transplanted with MSCs, elevated serum concentration of anti-inflammatory IL-10 was observed, while the levels of IL-6 and TNF-α, which are considered as promoters of liver fibrosis, were downregulated when compared to the other cell-administered groups and non-cell-treated but CCL_4_ challenged control [93].

Several preclinical rodent studies have clearly demonstrated the beneficial effects of human BM-MSCs (hBM-MSCs) in chemically induced liver fibrosis [94,95]. In one such study, Chang et al. [94] transplanted 1 × 10^6^ culture-expanded hBM-MSCs into CCl_4_-treated rats. At 4 weeks post-delivery, the levels of serum albumin and fibrinogen in the cell-treated group returned to normal levels, indicating restoration of specific liver functions. In addition, the incorporated hBM-MSCs expressed human α-fetoprotein, albumin, and cytokeratin-18, suggesting that transplanted cells can differentiate into albumin-secreting hepatocyte-like cells; hence, they contribute to the healing of the damaged liver [94]. Similarly, another study on a mouse model of CCl_4_-induced liver fibrosis showed that intravenous administration of hBM-MSCs at a dose of 5 × 10^5^ significantly reduced fibrosis levels 4 weeks after the completion of surgery [95]. Moreover, in the group of animals receiving hBM-MSCs, increased MMP-9 expression and decreased αSMA, TNFα, and TGFβ expression in liver tissue was observed. Although the mechanisms by which BM-MSCs reduced hepatic fibrosis have not been well established, the authors postulated that it might occur, in part, through enhanced expression of MMP-9, which is important for ECM remodeling [95].

Several studies have shown that ASCs or human UC-MSCs (hUC-MSCs) administered in rodent models of hepatic damage also led to reduced tissue fibrosis and provided restoration of liver function [94,95,96,97,98]. A study in which ASCs were first pretreated in the hepatogenic medium and then transplanted into rats with liver injury showed a decrease in circulating liver enzymes such as ALT and AST along with improved serum albumin level as compared with PBS controls [97]. In thioacetamide-induced chronic liver fibrosis in rats, transplantation of hASCs at a dose of 1 × 10^6^ by direct liver injection resulted in the recovery of organ function, as evidenced at 3 weeks by biochemical analysis of total bilirubin (TBIL), prothrombin time (PT), and albumin levels [96]. The authors found a significant decrease in liver fibrosis and inflammatory activity measured by metavir-based activity score in the ASC group. In addition, reduced expression of αSMA after 14 days and increased expression of MMP-9 in liver tissue at 7 and 14 days post transplantation were detected. Moreover, immunohistochemistry showed that hASCs injected directly into the liver differentiated into albumin- and α-fetoprotein-secreting liver-like cells as early as 1 week after transplantation [96]. Significantly, another group showed that ASCs were able to survive up to 4 months after engraftment into rat liver, and some of them acquired hepatocyte (asialoglycoprotein receptor ½ (ASGPR1/2)-positive) phenotype [98].

In the case of hUC-MSCs, it has been reported that administering them in a cirrhotic rat model at a dose of 1 × 10 ^6^ mitigated the profibrotic action of CCl_4_ [99]. As demonstrated in this work, histopathological fibrosis score at 4 weeks follow-up showed improvement in the structure of the cirrhotic liver. Additionally, a significant decrease in mRNA and protein levels of TGFβ, collagen type I, and αSMA were observed in liver tissue after 2 and 4 weeks [99]. Similar results were obtained by Tsai et al. [100], who transplanted 5 × 10^5^ hUC-MSCs into rats and, after 4 weeks, found a significant reduction in liver fibrosis, lower levels of serum glutamic oxaloacetic transaminase and glutamic pyruvate transaminase, and decreased protein expression of αSMA and TGFβ1 in the liver. However, engrafted hUC-MSCs were localized mostly in the hepatic connective tissue and did not differentiate into hepatocytes. Instead, these undifferentiated cells were able to secrete bioactive cytokines, including cutaneous T cell-attracting chemokine and leukemia inhibitory factor [100]. Overall, these data suggest that the effect of hUC-MSCs on reducing fibrosis might rely on bioactive factors released from the grafted cells rather than on their differentiation into hepatocytes. This concept was further supported by a study in which exosomes derived from hUC-MSCs were transplanted into a CCl_4_-induced mouse model of liver fibrosis [101]. The authors showed that 3 weeks after exosome delivery, liver fibrosis was significantly reduced. Similarly, the expression of *collagen I* and *III* mRNA and TGFβ1 protein decreased in liver tissue after 3 weeks. Notably, the results obtained from immunohistochemistry concerning E-cadherin, N-cadherin, and vimentin expression indicated that the exosomes contributed to the decline of the EMT process [101].

It should also be noted that there are studies reporting that MSCs might not be effective at providing improvements in hepatic fibrosis [102,103]. Carvalho et al. [102] investigated the effect of BM-MSCs administered at a dose of 1 × 10^7^ in rats with liver fibrosis induced via CCl_4_ injection and associated with an alcoholic liquid diet. They showed no improvement in serum biochemical markers of liver disease, including ALT and AST, or in collagen deposition in the liver at 1 and 2 months post transplantation [102]. Likewise, Mannheimer et al. [103] used a similar model of rats with liver damage that were injected through the portal vein with 1.6 × 10^7^ BM-MSCs isolated from cirrhotic rats. They found no significant differences in ALT and albumin blood levels between the cell-treated and placebo groups. Furthermore, no improvements were observed in portal vein diameter (PVD), liver parenchyma echogenicity, or collagen deposition. One explanation for the lack of efficacy of the therapy might be the use of BM-MSCs from cirrhotic rats, which may impact the functionality of MSCs [103].

Concerning clinical research, there have been several published stem cell-based trials that investigated the therapeutic effect and safety of MSCs in patients with liver fibrosis, mostly those with advanced cirrhosis [104,105,106,107,108,109,110,111,112]. The majority of trials indicated a clinical benefit of MSC transplantation in cirrhotic disease (Table 1). Indeed, a non-controlled phase I–II clinical trial with eight patients with end-stage liver disease (four with hepatitis B, one with hepatitis C, one with alcoholic, two cryptogenic) who received autologous BM-MSCs at approximately 3–5 × 10^7^ exhibited improved liver function verified by the model for end-stage liver disease (MELD) score, which decreased from 17.9 ± 5.6 to 10.7 ± 6.3 [108]. Moreover, decreased prothrombin complex, serum creatinine, and bilirubin was detected at 24 weeks follow-up. Importantly, no side effects of this cell therapy were demonstrated, and all patients reported subjective improvements beginning 2 months after transplantation [108].

In another controlled trial, Peng et al. [110] enrolled 53 patients with liver failure due to chronic HBV infection, who received autologous BM-MSCs through the hepatic artery (105 patients served as the control group). The results showed that the levels of ALT and TBIL, PT, and MELD score improved significantly after 2–3 weeks compared to patients in the control group. A follow-up study at almost 4 years showed no major differences in the mortality of patients and the incidence of hepatocellular carcinomas between patients with and without cirrhosis in the BM-MSC group [110]. Similarly, benefits were reported in a randomized controlled trial carried out on 20 patients with end-stage liver failure due to chronic HCV infection who underwent transplantation of autologous BM-MSCs stimulated to hepatic lineage (approximately 2 × 10^7^ hepatic lineage-committed cells). Compared to the control group, in patients who received cell therapy, parameters such as Child score, MELD score, fatigue scale, and performance status significantly improved at 6 weeks follow-up. Moreover, lower limb edema and serum albumin decreased in the cell-treated group relative to the control group [104].

Many similar beneficial effects were observed in two other clinical trials in which autologous BM-MSCs were transplanted into patients with liver cirrhosis induced by HCV [105,106]. In a separate phase II trial, 11 patients with alcoholic liver cirrhosis received two injections in the hepatic artery with 5 × 10^7^ autologous BM-MSCs, at weeks 4 and 8 [107]. Histological examination of liver biopsy specimens collected 12 weeks post transplantation showed improvement in 6 out of 11 patients (54.5%). The Child–Pugh score, which is a direct marker of hepatic fibrosis and the development of side effects, improved in 10 patients (90.9%). Furthermore, mRNA levels of *Tgfβ1*, *Col1*, and *αSMA* significantly decreased in liver tissue following BM-MSC transplantation. Importantly, no significant complications or side effects were reported [107]. Another controlled clinical trial was related to patients with decompensated liver cirrhosis who received hUC-MSCs at a dose of 5 × 10^5^ kg/body weight. Decreased hypogastric ascites volume and serum levels of laminin, procollagen III, COLIV, and hyaluronic acid (HA) were found, while the serum level of HGF was elevated [112]. On the other hand, there are also studies demonstrating a lack of efficacy of MSC therapy for the treatment of liver cirrhosis. Indeed, Mohamadnejad et al. [109] conducted a randomized placebo-controlled trial in which autologous BM-MSCs were peripherally infused into patients with decompensated cirrhosis. The results at 12 months post transplantation revealed no beneficial effect on the biochemical parameters of patients. Moreover, the absolute changes in MELD and Child scores, serum albumin, international normalized ratio (INR), serum transaminases, and liver volume did not differ significantly between the BM-MSC recipients and the placebo group [109].

Mesenchymal stem cells have emerged as a promising agent for the treatment of liver fibrosis and cirrhosis, mostly due to their immunomodulatory capacity and ability to differentiate into tissue-specific cells. Cell-based therapy seems to be extremely important because advanced fibrosis causes cirrhosis, for which liver transplantation is the only effective treatment. However, due to the limitations of liver transplantation, alternative therapeutic options are needed. However, there are some concerns about the efficacy and safety of using MSCs in patients with liver fibrosis, as these cells might show undesirable effects or even worsen the disease due to their profibrogenic potential [113,114].

### 3.3. Cardiac Fibrosis

Cardiac fibrosis is a common pathological condition related to heart injury and nearly all types of heart disease, including myocardial infarction (MI), coronary heart disease, hypertension, and genetic disorders associated with cardiomyopathies [115]. The mechanism underlying cardiac fibrosis is comparable to that in other organs. In brief, after heart injury, the death of cardiomyocytes triggers an inflammatory and fibrogenic response, leading to the formation of scar tissue, which preserves the structural and functional integrity of the myocardium (Figure 2) [116]. Regardless of the cause, fibrosis leads to cardiac tissue stiffness, contractile dysfunction, impaired myocardial function, and arrhythmogenicity, and subsequently, the condition progresses to heart failure [117]. Depending on the location and etiology, cardiac fibrosis can be divided into four types: reactive interstitial, replacement, infiltrative interstitial, and endomyocardial fibrosis (Figure 2) [118]. According to the World Health Organization’s list of the top 10 causes of death, heart diseases are the leading cause of death in the world. Since cardiac fibrosis is commonly associated with cardiovascular disease, considerable efforts are being devoted to the search for antifibrotic treatments. Researchers and clinicians are focused mainly on the inhibition of molecules that activate cardiac fibroblasts and affect the development of cardiac fibrosis.

The results of numerous studies with CTGF, galectin 3 (Gal-3), TGFβ, endothelin, MMPs, mineralocorticoid receptors, and the renin-angiotensin-aldosterone system as targets for antifibrotic therapy demonstrated attenuation of cardiac fibrosis and reduced ECM protein synthesis in different animal models [119,120,121,122]. However, these pharmacological therapies failed due to the occurrence of harmful side effects, such as adverse cardiac remodeling, gastrointestinal and liver dysfunction, and even death. Thus, effective pharmacotherapy for preventing or reversing cardiac fibrosis is presently unavailable. Therefore, novel approaches involving stem cell-based therapy have been introduced into the field of cardiovascular research.

Mesenchymal stem cells provide an attractive therapeutic approach for cardioprotection, particularly due to their ability to differentiate into cardiovascular cells in vivo and in vitro and by exerting immunomodulatory and angiogenic properties [123,124,125]. Moreover, the absence of cell surface histocompatibility complex (HLA) class II and T cell co-stimulatory molecules reduces the risk of transplant rejection, which makes MSCs useful in both autologous and allogeneic cell therapy [123,126,127]. The great majority of studies examined the therapeutic effect of BM-MSCs in the treatment of cardiac fibrosis. One such study showed that transplantation of BM-MSCs in mouse hearts after MI contributed to reduced fibrosis by modulating abundantly deposited ECM [128]. Collectively, the results of all of these studies demonstrate that transplantation of BM-MSCs or cardiac stem cells in a rat heart failure model resulted in reduced total collagen volume, decreased mRNA levels of *Col1* and -*3*, *TIMP-1*, and *TGFβ*, and expression of gelatinases in the myocardium [129,130,131,132]. There is also an increasing body of work supporting the idea that the effect of MSCs on fibrosis may be mediated through reduced inflammation. The results of a study conducted by Du et al. [133] showed that delivery of BM-MSCs into peri-infarct rat myocardium immediately after induction of MI led to inhibition of NF-kappa B activity, attenuated protein levels of TNF-α and IL-6, and increased expression of anti-inflammatory IL-10 in the myocardium [133]. Another study showed that the use of 3 × 10^6^ BM-MSCs in a rat model of MI reduced the levels of CD68-positive inflammatory cells and monocyte chemotactic protein-1 (MCP-1) in the myocardium at 3 weeks; thus, the cells contributed to improved cardiac function [134].

There is considerable evidence that MSCs have a therapeutic function in cardiovascular diseases primarily through paracrine action [135,136,137,138,139]. Kishore et al. [137] showed that transplanted bone marrow progenitor cells (BMPCs) had an antifibrotic effect by paracrine regulation of cardiac miRNAs in mice. Intramyocardially transplanted BMPCs at a dose of 1 × 10^6^ in *db/db* (diabetic) mice subjected to MI released HGF, which inhibited the miR-155-mediated pro-fibrosis response, leading to decreased mRNA expression of *Col1A1*, *Col3A1*, and *αSMA*, and concomitantly improved cardiac function [137]. Another study showed that conditioned medium from BM-MSCs markedly increased the mRNA expression of negative regulators for cell proliferation, such as *elastin* (*Eln*), *myocardin*, and *DNA-damage inducible transcript 3* (*DDIT3*) [138]. Moreover, there are data demonstrating that exosomes secreted by cardiac MSCs improved cardiac function by enhancing capillary density and cardiomyocyte proliferation in ischemic mouse myocardium [136].

Another study showed that exosomes from BM-MSCs are enriched with miR-22, and injecting them into ischemic hearts of MI mice significantly downregulated *Mecp2*, consequently diminishing apoptosis in ischemic myocardium and leading to a reduced fibrotic area [135]. Similarly, it has been shown that exosomes derived from rat BM-MSCs overexpressing GATA4 displayed antifibrotic properties in vivo, partially related to the high expression of miR-19a [139]. In addition, the administration of exosomes secreted from hypoxic cardiac stem cells resulted in reduced heart fibrosis in rats, while the administration of exosomes in TGFβ1-stimulated rat cardiac fibroblasts decreased mRNA transcription of profibrotic factors [140]. Accumulative data demonstrate that genetically engineered BM-MSCs that overexpress Akt, HGF, insulin growth factor 1 (IGF-1), miR-133, or stromal-cell-derived factor 1 alpha (SDF-1α) reduced myocardial fibrosis and restored cardiac function more efficiently than their non-engineered counterparts [131,141,142,143,144]. Of note, pretreating MSCs with melatonin, sildenafil, or anti-ischemic drugs (trimetazidine) or exposing the cells to hyperbaric oxygen (HBO) or even anoxic preconditioning enhanced their survival and led to amplified antifibrotic activity following myocardial implantation in vivo [124,145,146,147,148].

Because MSCs injected into infarcted hearts show low efficacy due to their low retention in the cardiac tissue, a variety of improvements were undertaken to enhance their survival rate. Indeed, encapsulating cells in injectable materials or loading them into porous scaffolds or hydrogels have been found to be innovative solutions to augments the therapeutic activity of transplanted cells [149,150,151,152]. The results of cumulative studies with the use of different scaffolds for MSC transplantation, including platelet-rich fibrin, microporous alginate-chitosan, co-polymers, and hyaluronan-based scaffolds, demonstrated that these biomaterials enhanced the antifibrotic effect of MSCs by increasing myocardial vascularization or reducing the degree of fibrosis in the scar area, thereby improving heart function after MI [149,150,151,152].

Embryonic stem cells, due to their ability to differentiate into functional cardiomyocytes represented by all types of specialized heart cells, including atrial-, ventricular-, sinus nodal-, and Purkinje-like cells, have been shown to exert a therapeutic effect in rodent models of cardiac fibrosis [153]. In a series of papers, Singla et al. [154,155,156] showed that mouse ESCs or their conditioned media improved cardiac function by multiple mechanisms. Upon transplantation into the post-MI myocardium, they both led to inhibition of cardiac myocyte cell death and enhanced activation of the Akt cell survival pathway in the myocardium. Their therapeutic effect was achieved through decreased MMP-9 signaling and increased levels of HGF and insulin growth factor 1 (IGF-1) in the heart [154,155,156]. Other studies have shown that transplantation of mouse ESCs overexpressing TIMP-1 significantly enhanced cardiac myocyte differentiation, leading to reduced cell apoptosis, increased Akt activity, and decreased MMP-9 in infracted myocardium compared with animals that received ESCs or control [157]. At the functional level, echocardiography showed that fractional shortening and ejection fraction (EF) were significantly improved in the group administered ESCs overexpressing TIMP-1 [157]. Furthermore, mouse and human ESCs lethally inactivated with irradiation injected into ischemic myocardial tissue of mice and rhesus macaque monkeys, respectively, were reported to result in improved myocardial function and decreased myocardial infarct size [158]. Interestingly, injection of mouse ESC-derived exosomes into infarcted mouse hearts augmented the survival of cardiac progenitor cells and stimulated the formation of cardiomyocytes in ischemic hearts. These improvements were probably mediated by the miR290–295 cluster, specifically miR-294, which highly enriched exosomes from mouse ESCs [159].

Based on the experimental success of using MSCs as a treatment for cardiovascular diseases in animal models, many clinical trials have been conducted to investigate the efficacy of different types of MSCs (Table 1) [160,161,162,163,164]. In detail, in a phase I/II randomized pilot trial (the POSEIDON trial), Hare et al. [127] tested the safety and effectiveness of autologous and allogeneic BM-MSCs in patients with ischemic cardiomyopathy. The cells were delivered by a transendocardial injection into 10 left ventricular sites in doses of 2 × 10^7^, 1 × 10^8^, and 2 × 10^8^ of each cell type. The administration of both cell types was safe and associated with low rates of serious adverse events in the 13-month follow-up. Following the cell transplantation, reduced scar size accompanied by decreased end-diastolic and end-systolic volume and sphericity index was observed. However, improvements in 6MWT and quality of life were associated only with autologous, not allogeneic, MSC therapy [127]. The same group conducted a randomized controlled trial in which they compared the safety and efficacy of autologous versus allogeneic BM-MSCs in patients with nonischemic dilated cardiomyopathy (NIDCM) [126]. The superior effect of allogeneic over autologous BM-MSC therapy was demonstrated at 12 months. Indeed ejection fraction EF and 6MWT increased significantly in the allogeneic group compared to the autologous group. In addition, endothelial function was improved only in the group treated with allogeneic BM-MSCs, and TNFα suppression was also greater in this group. Consistently, the occurrence of serious adverse events was 28.2% in allogeneic BM-MSC recipients and 63.5% in the autologous group [126]. Collectively, these findings provide evidence of a clinically relevant effect of allogeneic BM-MSCs of greater magnitude than that observed using autologous cells.

Florea et al. [162] conducted a study (the TRIDENT study) using allogeneic human BM-MSCs in two doses (2 × 10^7^ and 1 × 10^8^) administered via transendocardial injection to patients with ischemic cardiomyopathy. Twelve months after MSC administration, no serious treatment-related adverse events were observed. The allogeneic MSC therapy improved cardiac function, with scar size reduction in both groups and an increase in ejection fraction only in the group that received the higher dose [162]. These findings underline the importance of using the proper cell dose in response to cell therapy.

Besides BM-MSCs, the safety and efficacy of other cell types, including ASCs, UC-MSCs, and Wharton’s jelly-derived MSCs, were evaluated in clinical trials [165,166,167]. Direct intramyocardial injection of ASCs and intravenous infusion of UC-MSCs or Wharton’s jelly-derived MSCs did not cause any complications or serious adverse events in patients with heart failure. Moreover, increased myocardial viability, enhanced function of the left ventricle, and improved quality of life were reported in patients treated with MSCs [165,166,167]. Although these clinical trials reported some beneficial effects of the treatment of cardiac failure, none of them indicated that stem cell transplantation entirely reduced heart fibrosis. There are a few ongoing registered clinical trials (https://clinicaltrials.gov/; 16 September 2021) concerning the use of MSCs in the treatment of cardiac fibrosis, which so far have not provided any results.

Despite many pieces of evidence indicating the advantages of MSCs in heart failure treatment, cell-based therapy still faces some challenges, such as the poorly targeted migration, the low rate of MSC differentiation into cardiomyocytes, and the low survival rate. On the other hand, the use of ESCs, despite their documented effectiveness in the treatment of cardiac fibrosis in animals, is limited by an ethical controversy related to their source of origin in humans, immunogenic potential, risk of cancer formation (teratomas formed in approximately 50% of rats injected with ESCs; [168], and low rate of differentiation into cardiomyocytes (cardiomyocytes usually represent less than 1% of the total cells in cell cultures [161]). Therefore, elaborate protocols may be needed for immunosuppressive therapy in order to reduce the risk of cell rejection. In vitro differentiation of ESCs before transplantation might be an option to increase the production of cardiac cells and avoid tumor development post-delivery.

### 3.4. Renal Fibrosis

Renal fibrosis is considered as a common final consequence of a variety of chronic renal diseases, including chronic glomerulonephritis, diabetic nephropathy, hypertensive nephropathy, and chronic renal allograft injury [169]. All of the functional compartments of the kidney can be affected by fibrosis, which is specifically termed interstitial fibrosis in the tubulointerstitium, glomerulosclerosis in the glomeruli, and arteriosclerosis or perivascular fibrosis in the vasculature [170,171].

The aging population and the rising prevalence of diabetes, obesity, atherosclerosis, and hypertension increase the incidence of kidney diseases, and consequently, renal fibrosis. In turn, renal fibrosis leads to eventual end-stage renal disease, organ failure, and the need for renal replacement therapy (i.e., dialysis or transplantation) (Figure 2) [172]. Inhibiting renal fibrosis from progressing is crucial in order to prevent progressive deterioration of kidney function, and it could avoid the final solution in the form of renal replacement therapy. In recent years, many efforts have been made to identify mediators and targets for renal fibrosis therapy. The results of many animal studies have revealed efficiency in reducing renal fibrosis and improved kidney function after the inhibition of molecules directly involved in the development of fibrosis [171,172,173,174]. However, such therapies failed in clinical trials due to an inappropriate balance between antifibrotic efficacy and adverse effects [173,174]. In the face of the lack of an effective method of treating renal fibrosis, the employment of stem cell therapy represents a promising treatment strategy.

A large body of data has demonstrated the therapeutic effects of BM-MSCs in rodent models of renal fibrosis [175,176,177,178,179,180,181,182,183,184,185,186,187,188,189,190,191]. Administration of BM-MSCs into renal parenchyma or intravenous delivery led to reduced interstitial fibrosis reflected by decreased expression of ECM components and vimentin, reduced MMP-2 activity, and inhibited TGFβ/SMAD signaling in the damaged kidney [175,176,177,178]. Several studies showed that the renal expression of E-cadherin was upregulated, while the expression of αSMA and desmin were downregulated after BM-MSC injection [175,177,178,179]. Additionally, BM-MSCs were found to attenuate renal inflammation by downregulating inflammatory mediators such as c-c motif chemokine ligands (CCL)-4, -7, -19, IFN-α/β, TNFα, IL-1β, and IL-6 [175,177,180]. There is also evidence that one of the mechanisms by which MSCs might exert their antifibrotic activity in the kidney involves regulation of the cell cycle of tubular epithelial cells (TECs). Generally, injury to TECs results in the arrest of cells in the G2/M phase of the cell cycle. This cell cycle arrest mediates fibrosis by excessive production of profibrotic factors, including TGFβ, epidermal growth factor (EGF), TNFα, nuclear factor kappa B (NF-κB), lipocalin 2 (NGAL), and hepatitis A virus cellular receptor 1 (KIM-1) [171,181,182]. Zhu et al. [183] demonstrated that transplanting human ASCs into mice with induced acute kidney injury (AKI) caused a significant decrease in the number of TECs arrested in G2/M, correlated with reduced pathological renal damage [183].

A growing body of evidence indicates that BM-MSCs mediate their effects mainly through paracrine mechanisms [184,185]. Extracellular vesicles (Evs) derived from MSCs have been shown to contribute to kidney repair [184,185,186,187]. Several studies on rodents demonstrated attenuation of renal fibrosis after injection of BM-MSC-conditioned medium or BM-MSC-derived Evs, through their involvement in inhibiting apoptosis, stimulating tubular epithelial cell proliferation, and decreasing the expression of COLI, αSMA, TGFβ1, TNFα, TIMP-1, MMP-3, and snail family transcriptional repressor 1 (SNAI1) in the kidney [184,185,186,187]. Similarly, the administration of ASCs or ASC-derived Evs to damaged pig or rodent kidneys caused reduced renal fibrosis and decreased inflammation [188,189,190]. Additionally, such therapy has been shown to decrease the occurrence of EMT, inhibit TGFβ/SMAD signaling and the renin-angiotensin system, and induce a shift in the macrophage phenotype from inflammatory to reparative in renal tissue [183,188,189,190]. Likewise, delivery of 2 × 10^7^ microparticles produced by kidney-derived MSCs into mice with unilateral ureteral obstruction, a model of tubulointerstitial scarring, inhibited the infiltration of inflammatory cells and suppressed tubulointerstitial fibrosis, as demonstrated by a decrease in F4/80- and αSMA-positive cells [191].

A significant reduction in renal fibrosis in mice was also demonstrated after administration of ASCs overexpressing glial cell line-derived neurotrophic factor (GDNF) or exosomes derived from GDNF-transfected ASCs [192]. This effect was linked to suppression of inflammation and activation of the sirtuin 1/ nitric oxide synthase (SIRT1/eNOS) signaling pathway in renal tissue [192]. Several studies also reported that administration of BM-MSCs overexpressing HGF, ACE2, vascular endothelial growth factor (VEGF), or miRNA-let7c or in combination with antifibrotic serelaxin effectively reduced renal fibrosis in a rat model [193,194,195]. Indeed, such an approach was effective at inhibiting fibrosis, manifested by decreased expression of αSMA, COLI, COLIV, MMP-9, and FN, inhibition of TGFβ signaling, and upregulation of MMP-2 expression in renal tissue [193,194,195,196,197]. Additionally, recent studies demonstrated that MSC culture in vitro and preparation prior to transplantation might affect the therapeutic properties. Indeed, two independent studies showed that the use of BM-MSCs pretreated with melatonin or IFNγ ameliorated interstitial fibrosis compared with control groups [198,199]. Similarly, amniotic fluid-derived stem cells (AFSCs), which exhibit characteristics of both ESCs and MSCs, preconditioned with GDNF, were shown to abrogate the degree of renal interstitial fibrosis in mice [200]. Intravenous delivery of 3.5 × 10^5^ GDNF-AFSCs suppressed oxidative stress and inflammation, repaired renal microvessels, and relieved tissue hypoxia and mitochondrial damage in the kidney [200].

The results of both in vitro studies and preclinical in vivo investigations conducted on rodent models of renal fibrosis demonstrated that administration of MSCs from the umbilical cord and placenta or their conditioned media was effective at inhibiting renal fibrosis [201,202,203,204,205,206]. This effect has been linked to decreased expression of COLI, FN, αSMA, CTGF, and proinflammatory cytokines in renal tissue. Moreover, inhibition of the EMT process or TGFβ/Smad and TLR4/NF-κB signaling or reinforcement of the Akt signaling pathway has been demonstrated in kidneys [201,202,203,204,205,206].

Although human ESCs have the ability to differentiate into aquaporin (AQP) 1- or AQP 2-positive cells showing morphological and functional features typical of specific renal cells, there is a limited number of studies concerning the impact of ESCs on renal fibrosis [207,208]. In work performed by Geng et al. [209], murine ESCs were loaded into gelatin microcryogels, and the structures were packed into pedicled greater omentum flaps in a rat model of chronic kidney disease. At 12 weeks after transplantation, reduced glomerulosclerosis and tubular injury were found. Moreover, the levels of plasma creatinine and urea nitrogen were decreased in ESC-treated animals compared to the control group [209]. In a separate study, De Chiara et al. [210] examined the impact of tubular-like cells (GTCs) that arise from germline cell-derived pluripotent stem cells (GPSCs) on mice with ischemic renal injury. They found that the cell treatment reduced cortical damage, tubular apoptosis, and renal oxidative stress while it upregulated tubular expression of the antioxidant enzyme hemeoxygenase-1. Moreover, at 6 weeks, kidneys of the mice that received GTCs showed less fibrosis, and reduced inflammatory infiltrate compared to kidneys of vehicle-treated counterparts [210]. Besides ESCs, iPSs were also shown to have the capacity to differentiate into renal lineages or cells that exhibit a renal-like phenotype and gene signature [211,212]. A recent study showed that human iPSCs (hiPSCs) generated from peripheral blood, along with renal progenitor cells (RPCs), differentiated from them, reduced interstitial fibrosis, tubular atrophy, and glomerulosclerosis in a rat model of chronic kidney disease [213]. Caldas et al. [214] used a similar experimental rat model of kidney disease and injected into 0.5  ×  10^6^ iPSCs derived from rat skin fibroblasts into renal parenchyma. They found elevated serum creatinine, reduced glomerulosclerosis, and decreased macrophage infiltration. However, histopathological analysis of liver specimens revealed tumors with characteristics of nephroblastoma, suggesting that although iPSCs seem to be encouraging as cell therapy, they carry a risk of Wilms’ tumor development [214].

Despite the experimental success of MSCs in the treatment of renal diseases in animal models and the results from a limited number of human clinical trials demonstrating the safety and feasibility of MSC-based kidney therapy, the efficacy of the studies remains controversial (Table 1). Swaminathan et al. [215] conducted a phase II randomized placebo-controlled trial to determine the safety and efficacy of allogeneic MSCs in reducing the recovery time from AKI after cardiac surgery. Intra-aortic administration of MSCs at a dose of 2 × 10^6^ cells/kg body weight was safe and well tolerated but did not markedly improve renal function or patient mortality. Further, MSC transplantation did not decrease the time to recovery of kidney function [215]. Similarly, the results of another clinical study in which autologous BM-MSCs at a dose of 2 × 10^6^ cells/kg body weight were given to patients with autosomal-dominant polycystic kidney disease (ADPKD) revealed no cell-related adverse effects at 12 months [216]. Concomitantly, MSC infusion did not induce any significant changes in estimated glomerular filtration rate (eGFR) or reductions in serum creatinine compared to baseline in all patients [216].

Saad et al. [68] investigated the role of intravenously infused autologous ASCs at doses of 1 × 10^5^ and 2.5 × 10^5^ for the treatment of atherosclerotic renovascular disease (RVD). They found that ASC infusions were well tolerated by the patients. Three months after cell transplantation, increased cortical perfusion and renal blood flow were reported. Increased kidney perfusion was accompanied by decreased fractional tissue hypoxia and stabilization of the glomerular filtration rate [68]. In a separate non-randomized and placebo-free phase I clinical trial, Alatab et al. [66] treated patients receiving peritoneal dialysis with autologous ASCs at a dose of 1.2 ± 0.1 × 10^6^ cells/kg. While hematological and systemic biochemical parameters were stable over 24 weeks, a significant change in the ASC group was decreased body mass index (BMI), which probably resulted from the decrease in the degree of edema due to the increased ultrafiltration rate [66]. The results of other studies verifying the validity of autologous MSCs (https://clinicaltrials.gov/, accessed 16 September 2021) in patients suffering from chronic kidney disease, renovascular hypertension, or occlusive kidney disease have been not published yet. Currently, among all registered clinical trials concerning the use of MSCs for the treatment of kidney diseases (https://clinicaltrials.gov/, accessed 16 September 2021), only a few focus on the treatment of renal fibrosis. To date, none of these studies have provided results.

The standard methods of treating renal fibrosis are limited; however, the use of stem cells seems to have potential as a therapeutic approach for renal fibrosis-related diseases. In particular, MSC-based therapy, which has been demonstrated as being safe and feasible, has vital potential to improve renal function and patient mortality. Further studies should be conducted to improve the long-term therapeutic activity of transplanted cells.

### 3.5. Uterine Fibrosis (Asherman Syndrome)

Asherman syndrome (AS) is a complex gynecological disorder characterized by intrauterine adhesions (IUAs) that lead to defective function of the endometrium. Surgical procedures that disrupt the endometrial basalis layer, such as excessive postpartum or post-abortion curettage and frequent hysteroscopic surgery, as well as recurring infections, are the main factors responsible for the development of IUAs (Figure 2) [217,218]. Endometrial trauma causes molecular changes that may lead to fibrosis. As a result, the endometrium stops responding to hormonal stimulation, and the uterine cavity becomes obstructed, leading to the creation of IUAs [217]. Severe cases of AS lead to abnormal uterine bleeding, amenorrhea, hypomenorrhea, chronic pelvic pain, abnormal placentation, recurrent miscarriages, or even infertility. So far, the main therapy for AS relies on surgical intervention, with hysteroscopic surgery performed to re-establish a normal uterine cavity and restore uterine function [219]. However, traumatized endometrium tends to develop post-surgical intrauterine adhesions, so the actual challenge in handling AS is to prevent or minimize the development of new adhesions [220]. Therefore, post-surgical methods, including inserting a balloon or other type of intrauterine device or creating a protective lining with HA, have been applied to provide a physical barrier inside the uterus that prevents the occurrence of new adhesions. Moreover, to promote the healing process, estrogen and antibiotic treatment are also recommended [221,222,223].

The development of IUAs involves the destruction of the basalis layer of the endometrium, which is rich in somatic stem cells responsible for its cyclic regeneration. Therefore, stem cell transplantation might be a promising approach for the reconstruction of the stem cell pool, which in turn might contribute to endometrial renewal and AS resistance. Indeed, several lines of evidence indicate that transplanting MSCs to the endometrium contributes to the renewal of this tissue [224,225]. Carvelló et al. [226] showed that transplanted human CD133^+^ BM-MSCs in the uterine horn in an AS mouse model successfully engrafted around blood vessels and induced proliferation of surrounding cells via paracrine factors such as thrombospondin 1 (Thbs1) and IGF-1 [226]. A study by Alawadhi et al. [224] also conducted on a mouse model of AS demonstrated that transplantation of 1 × 10^7^ BM-MSCs improved fertility. In the BM-MSC transplant group, 9 of 10 mice conceived, whereas, in the non-transplanted control group, only 3 of 10 females were pregnant [224].

A recent report by Çil et al. [67] showed that a single dose of ASCs in female rats with experimentally induced AS maintained epithelial integrity in the endometrium, reduced leukocyte infiltration and fibrosis, and increased vascular proliferation. Interestingly, similar improvements were observed in mice that received combined therapy consisting of ASCs and oral estrogen. Of note, the authors assumed that the therapeutic effect might be mediated through IGF-1 and VEGF [67]. Interestingly, current data show that menstrual blood is a noninvasive and easily available source of endometrial MSCs (eMSCs) [227]. Domnina et al. [227] performed functional studies in which adult female Wistar rats received a suspension of human eMSCs, human eMSCs spheroids, or rat BM-MSCs via the intravenous or intrauterine route (eMSC spheroids were transplanted into the uterus). Three estrous cycles after AS induction and the delivery of cells, the females were mated with males, and finally, the number of pregnant animals and their litter size were recorded. It was found that the number of pregnant females significantly increased after stem cell administration compared to the PBS-treated group. A higher pregnancy rate was achieved with intrauterine than intravenous administration of cells. Notably, transplantation of eMSC spheroids produced a superior effect, manifested by the highest conception rate and the highest number of pups born [227].

Functional restoration of endometrium in a mouse AS model was shown to be mediated by human amniotic mesenchymal stromal cells (hAMSCs) [228]. Besides increases in the pregnancy rate and the number of fetuses upon hAMSC administration, the authors found several post-transplanted changes, including increased microvessel density and elevated expression of VEGF, proliferating cell nuclear antigen (PCNA), estrogen, and progesterone receptors, indicating endometrial repair responsible for the restoration of endometrial function [228].

The efficacy of MSCs for AS treatment has also been demonstrated in clinical research. (Table 1). Santamaria et al. [229] showed that autologous CD133^+^ BM-MSC administration in conjunction with hormonal replacement therapy affected patients with IUA. At the third month after treatment, increased epidermal thickness and neoangiogenesis and prolonged duration of menses were shown. Notably, 3 out of 16 patients became pregnant spontaneously, resulting in birth, while 7 positive pregnancies were obtained after 14 embryo transfers [229]. These findings were further supported by Singh et al. [225], who showed that transplantation of human BM-MSCs into women with AS led to a significant increase in endometrial thickness and resumption of menses in a majority of amenorrheic patients and may have allowed 3 out of 25 patients to spontaneously conceive, with positive pregnancy outcomes [225].

In a recent paper, Lee et al. [230] showed the results of a trial in which autologous SVFs at a dose of 4.6 ± 0.7 × 10^6^ were transplanted into the uteruses of six infertile women with severe AS. They found that SVF delivery followed by estrogen hormone therapy increased endometrial thickness from 3 mm to around 6.9 mm. After the combined therapy, five of the women had an embryo transfer; among them, one woman conceived but aborted spontaneously at 9 weeks gestation [230]. Another group demonstrated a therapeutic effect mediated by allogeneic UC-MSCs supported by a collagen scaffold transplanted into the uterine cavities of patients with AS-related infertility [231]. In agreement with other studies, the authors revealed that the average endometrial thickness improved at 3 months, and, importantly, there was a decrease in intrauterine adhesion scores compared to those before the treatment. In endometrial biopsies, the expression of estrogen receptor alpha (ERα), vimentin, Ki67, and von Willebrand factor increased, suggesting post-transplantation improvement in endometrial proliferation, differentiation, and neovascularization. Even more important, by the end of the 30-month follow-up period, 10 out of 26 patients had become pregnant, and 8 of them had positive pregnancy outcomes [231]. Similarly, autologous menstrual blood-derived stromal cells (menSCs) were demonstrated to improve endometrial thickness in women with severe AS, and notably, two of the four patients who underwent frozen embryo transfer conceived successfully, while one patient had a spontaneous pregnancy after a second menSCs transplantation [232].

Based on the above studies, MSCs exhibit a positive treatment effect in women with AS. The effects were reflected by morphological improvement of the endometrium, and functional enhancement was manifested by the resumption of menstruation and increased fertility outcomes of infertile AS women. Certainly, AS prevents implantation of the blastocyst and impairs the blood supply to the uterus and early fetus, and finally, the disease results in recurrent miscarriage or infertility. Although AS is not fatal, its effects on the psyche and quality of life of women make further research on stem cell-based therapy extremely important. Clinical trials have demonstrated the initial safety and effectiveness profiles of different types of stem cells that might provide potential options for treating women with severe AS.

### 3.6. Skin Fibrosis

Fibrotic skin disorders, although of unknown etiology, share a similar set of abnormal processes manifested by the presence of activated fibroblasts and excessive deposition of ECM [233]. The development of fibrotic skin lesions, such as in SS mainly occurs in association with metabolic and immunological disorders or can arise in response to dermal injury that might lead to the formation of hypertrophic scars or keloids (Figure 2) [233]. Experimental data on rodents showed that dermal fibrosis can be induced by local administration of drugs and chemicals such as bleomycin, vinyl chloride, or hypochlorous acid (HOCl) [234,235,236]. Moreover, there are several established genetic mouse models with a fibrotic skin phenotype, including fibrillin-1 mutant tight skin mice (*Tsk*), transgenic animals that include those that express a kinase-deficient type II TGFβ receptor, overexpress Wnt 10b, or are deficient in caveolin [236,237].

The prototype of fibrotic skin diseases is scleroderma, which can be classified into localized (LSc, morphea) and systemic (SS) types. Systemic sclerosis can be further divided into two clinical subtypes: limited cutaneous (lcSS), with fibrotic skin changes present in fingers, hands, and face, and diffuse cutaneous (dcSS), which initiates in fingers and hands and gradually spreads to limbs and trunk [233]. SS is a chronic multisystem disorder manifested by autoimmunity, microvascular dysfunction, and fibrosis of the skin and internal organs, including lungs and kidneys. The disease more frequently affects women than men and causes high mortality, with a survival rate between 34% and 73% [238]. The clinical features of SS primarily represent consequences of vascular impairment and ischemia, such as Raynaud’s phenomenon and digital ulcers [233]. Despite collagen overproduction, the pathophysiology of SS is characterized by damage to small vessels and dysregulation of the humoral and cellular immunity, reflected, at least in part, by the production of autoantibodies to nuclear, nucleolar, and cytoplasmic antigens as well as endothelial cells. Accordingly, emerging data demonstrate that the sera of individuals with scleroderma have antibodies against centromere, Scl-70/topoisomerase I, anti-fibrillarin/anti-U3 nucleolar antigens RNA polymerase I–III, and those that block MMP-1 and MMP-3 [233,239].

SS is characterized by fibrotic lesions that not only develop in the skin but also involve internal organs, and the disease is life-threatening, intractable, and resistant to standard immunosuppressive therapy. In addition to functional challenges of affected organs, cutaneous symptoms are often associated with pain and with psychological and esthetic distress and cause body image dissatisfaction. Importantly, many other scleroderma-like conditions, including scleredema, eosinophilic fasciitis (Shulman’s disease), porphyria cutanea tarda, diabetic stiff-hand syndrome, lichen sclerosis, and graft-versus-host disease (GVHD), show distinct cutaneous manifestations, pathological skin histology, and systemic implications (Figure 2). It should also be emphasized that the most common manifestation of cutaneous fibrosis is scarring that occurs after injury, which is a natural consequence of the wound healing process [240]. In contrast to mammalian fetuses, which heal cutaneous wounds without scars via regeneration, regular wound healing in adult mammals results in scar formation [241,242]. Overscarring is a frequent wound healing-associated fibrotic disorder that is clinically recognized as hypertrophic scars or keloids [243]. Although both generate excessive scar tissue, keloids are characterized by their extensive growth beyond the original wound site, while hypertrophic scars are raised but remain within the confines of the initial wound border [243].

Given that fibrotic skin diseases are complex, chronic, and heterogeneous and the evaluation of therapy is difficult, effective treatments have not been established so far. Nevertheless, among potential systemic antifibrotic therapies under investigation, those that employ MSCs have emerged as among the most promising [244,245,246,247,248].

Recent studies on HOCl-induced mouse models of SS revealed that systemic infusion of allogeneic BM-MSCs at a dose of 2.5 × 10^5^, 5 × 10^5^, or 10^6^ cells led to a reduction in inflammatory, oxidative, and fibrotic processes in skin and lungs [246]. Delivery of the cell concomitantly with HOCI resulted in 25%–30% lower skin thickness in the BM-MSC-treated group at day 21 post treatment. In addition, at 42 days, decreases in both collagen content and transcript levels of *Col1*, *Col3*, *Tgfβ1*, and *αSma* in the skin were observed, and the effect was inversely proportional to the cell dose. Notably, the second infusion of cells at day 21 led to further inhibition of skin thickening progression from days 28 to 42. Moreover, a significant reduction in the expression of mRNA of *Col1*, *Col3*, *Tgfβ1*, and *αSma* in the skin and *Col3* and *Tgfβ1* in the lung was detected [246]. This study indicated that not only the cell dose but also the time of cell injection may be an important factor controlling the efficacy of cell-based therapy. In a separate study, Chen et al. [245] indicated that systemic administration of allogeneic mouse BM-MSCs or BM-MSC-derived exosomes ameliorated autoimmune and dermal phenotypes of fibrosis in *Tsk*/^+^ mice through miR151-5p transfer to recipient cells. Specifically, in terms of skin, reduced hypodermal thickness was found. This effect was accompanied by a significant improvement in the bone formation rate, rescuing osteopenia in *Tsk*/^+^ mice. It was indicated that the therapeutic effect was achieved via IL-4 receptor alpha (IL4Rα) and decreased mTOR signaling gene expression in *Tsk*/^+^ BM-MSCs [245]. In a similar study, Akiyama et al. [244] demonstrated that BM-MSCs reduced hypodermal thickness and exerted an immunomodulatory effect via FAS/FASL-mediated apoptosis of T cells.

To evaluate the therapeutic effect of ASCs in a systemic model of scleroderma, Rubio et al. [248] employed a murine model of BLM-induced SS. As demonstrated in this work, intravenous ASC treatment at a dose of 5 × 10^5^ cells prevented the development of fibrosis in the lung and skin. Regarding skin, the therapeutic effect was evidenced by well-arranged collagen fibers parallel to the epidermis that resembled the collagen network observed in healthy, saline-treated control mice [248]. ASC administration attenuated the impairment in wound healing in BLM-treated mice, reflected by a significant reduction in total wound size by 58.8% as compared to the control group. The ASC-primed improvement in wound healing resolution was associated with decreased expression of miR-199–3p and upregulation of its corresponding target caveolin 1 in both lung and skin wounds [248]. These findings support results reported by other authors and indicate that one mechanism by which MSCs may regulate fibrotic pathways is by modulating miRNA expression in target tissues [245,248]. Other studies demonstrated that ASC administration was sufficient to attenuate BLM-induced scleroderma by suppressing the infiltration of CD4^+^ and CD8^+^ T cells and macrophages into the dermis, but it also reduced the frequency of CD4^+^ T cells and effector B cells in the spleens of SS mice [247]. Moreover, there was a decrease in mRNA expression of *Col1a2* and profibrotic cytokines such as *IL-6* and *IL-13*, suggesting an immunomodulatory and anti-inflammatory effect of ASCs in fibrotic skin [247].

To test the therapeutic potential of MSCs in wound healing-associated fibrosis, Domergue et al. [249] created a humanized animal hypertrophic scar model by delivering healthy human split-thickness skin grafts into the backs of immunocompetent nude mice. Next, at week 7 after graft implantation, human SVF suspension containing 1 × 10^6^ of hASCs, 1 × 10^6^ of cultured hASCs, or PBS used as a control was subcutaneously injected into the scar. The follow-up study revealed a reduction in hypertrophic scarring in the SVF- and hASC-treated group [249]. The reduction in post-wound skin thickness was more significant in the hASC-treated group than in the cohort that received SVFs. This effect corresponded with decreased collagen content in skin specimens. Moreover, cell-treated mice had higher mRNA levels of antifibrotic markers *TGFβ3* and *HGF*, elevated expression of *MMP-2*, and a higher *MMP-2/TIMP-2* ratio in hypertrophic scars, reflecting the remodeling activity contributing to fibrosis resorption [249]. A similar model, in which fragments of human keloid tissue were subcutaneously implanted into skin pockets created on the dorsal skin of athymic mice, was used to investigate whether the secretome of hASCs could provide a strategy against keloid formation [250]. Given that one mechanism by which MSCs exert their effect on a target tissue is paracrine secretions, in the aforementioned study, conditioned medium from ASCs (ASC-CM) was injected within keloid xenografts. At 4 weeks post treatment, increased keloid shrinkage along with better-organized collagen fibers was found in the ASC-CM-treated group. In addition, mice exposed to ASC-CM showed a reduced presence of CD31- and CD68-positive cells in keloid tissue [250]. Together, these results demonstrate the therapeutic potential of hASCs for clinical application in the treatment of hypertrophic scars and keloids.

Several clinical studies demonstrated the possible benefits of HSC and MSC (mostly SVF/ASC) therapy in SS patients (Table 1). In a recent paper, Henrique-Neto et al. [251] reported the results of a longitudinal study that included 70 adult SS patients with a severe SS profile who received autologous HSCs at a mean dose of 5.63 (3.21) × 10^6^ CD34^+^ cells/kg body weight. The most clinically detectable outcome of HSC administration at 5 years post transplantation was improved skin thickness, assessed by modified Rodnan’s skin score (mRSS). Of note, enhanced pulmonary function (forced vital capacity and diffusing capacity of carbon monoxide) was also observed [251]. These data are consistent with previous results from three randomized controlled trials that demonstrated the superiority of autologous HSC transplantation over the standard cyclophosphamide treatment: American Scleroderma Stem Cell versus Immune Suppression Trial (ASSIST, phase II) [252], Autologous Stem Cell Transplantation International Scleroderma Trial (ASTIS, phase III) [253], and Scleroderma: Cyclophosphamide or Transplantation (SCOT, phase II) [254].

Preclinical and clinical investigations have demonstrated promising therapeutic effects of fat grafting or autologous SVF/ASC-based therapy for patients with cutaneous manifestations of SS (Table 1). In the initial case report, patients with cutaneous manifestations of SS received a local injection of ASCs at a dose of 4 × 10^6^ to 8 × 10^6^ suspended cells in HA solution. Over 1 year of follow-up, all individuals exhibited arrest of local disease [255]. Specifically, four out of six patients showed dyschromia regression (67%), five patients showed improved skin softening (83%), four patients showed better sensitivity (67%), and one patient showed reduced erythema (17%). The study was one of the first to demonstrate a beneficial effect of locally implanted ASCs in patients with severe fibrosis. The first open phase I clinical trial was conducted among 12 SS patients with impaired hand function who received an injection of 3.76 ± 1.85 × 10^6^ autologous SVFs into each finger [256]. Preliminary assessment at 6 months post transplantation revealed reduced pain, increased grip strength, reduced finger circumference, and an average reduction in Raynaud’s severity by 67.5%. Moreover, significant decreases in dystrophic capillaries and vascular suppression scores were observed [256].

There are currently data demonstrating that besides enzymatically isolated SVFs/ASCs, an autologous lipoaspirate rich in multipotent stem cells can also be used as a cell-based therapy for SS [257,258]. As presented by Almadori et al. [257], the lipoaspirate injected directly into fibrotic oro-facial tissues of SS patients significantly improved mouth function and facial volumetric appearance. Similarly, another report showed that fat grafting into patients with advanced SS-related perioral thickening and mouth opening limitation resulted in a significant increase in interincisal distance and oral perimeter along with improved neovascularization [258].

The studies described above provide encouraging and supportive data for the use of MSCs, in particular SVFs or ASCs, as a vital therapeutic strategy against SS. Nonetheless, the results of larger, randomized double-blind and placebo-controlled trials are needed to obtain a meticulous clinical evaluation of MSCs.

**Table 1:** Clinical trials of stem cell therapies in fibrotic diseases in lungs, liver, heart, kidney, uterus, and skin.

Organ	Disease	Stem Cell Type	Number of Cells	Delivery Route	Effects	References
Lung	Idiopathic pulmonary fibrosis	Allogeneic BM-MSCs	2 × 10^8^ cells/infusion (4 infusions in 3 month intervals, total 1.6 × 10^9^)	Intravenous	Improved lung function based on FVC, DLCO, and 6MWT	[82]
	Idiopathic pulmonary fibrosis	Allogeneic BM-MSCs	2 × 10^7^ or 1 × 10^8^	Intravenous	Higher cell dose alleviated fibrosis progression	[83]
	Idiopathic pulmonary fibrosis	Allogeneic BM-MSCs	2 × 10^7^, 1 × 10^8^, or 2 × 10^8^	Intravenous	Improved lung function assessed by 6MWT at 36 weeks	[84]
Liver	Alcoholic cirrhosis	Autologous BM-MSCs	5 × 10^7^/injection (2 injections at study weeks 4 and 8)	Intra-arterial (right hepatic)	Histological improvement of liver biopsy based on Laennec fibrosis scoring system; decreased collagen deposition, mRNA expression of TGFβ1, Col1, and αSMA in liver biopsy, and MELD score; improved Child–Pugh score	[107]
	Hepatitis C-induced liver cirrhosis	Autologous BM-MSCs	1 × 10^7^	Intrasplenic injection	Improved liver function assessed by decreased TBIL, AST, ALT, PT, and INR levels and increased albumin and PC levels	[105]
	Hepatitis C-induced liver cirrhosis	Autologous BM-MSCs	1 × 10^6^/kg	Intravenous	Decreased jaundice, lower limb edema, MELD score, and serum creatinine level; improved encephalopathic manifestation, ascites, serum bilirubin, and albumin levels	[106]
	End-stage liver failure due to chronic hepatitis C	Autologous BM-MSCs	2 × 10^8^	Intrasplenic or intrahepatic injection	Improved liver function based on MELD and Child scores, fatigue impact scale, and performance status	[104]
	Liver failure caused by hepatitis B	Autologous BM-MSCs	N/A	Intra-arterial (hepatic)	Improved liver function assessed by ALB, TBIL, and PT levels and MELD score	[110]
	Liver cirrhosis	Autologous BM-MSCs	3–5 × 10^7^	Intravenous (peripheral or portal vein)	Improved liver function assessed by MELD score and decreased prothrombin complex, serum creatinine, and bilirubin at 24 weeks	[108]
	Liver cirrhosis	Autologous ASCs	3.3 × 10^5^ or 6.6 × 10^5^ cells/kg	Intra-arterial (hepatic)	Increased concentrations of serum HGF, IL-6, IL-18, M-CSF, and MIF at 1 day post treatment	[111]
	Decompensated liver cirrhosis	Allogeneic UC-MSC	5 × 10^5^ cells/k	Intravenous	Decreased hypogastric ascites volume and serum levels of plasma laminin, procollagen III, COLIV, and HA	[112]
Heart	Ischemic cardiomyopathy	Allogeneic BM-MSCs	2 × 10^7^, 1 × 10^8^	Transendocardial injection	Reduced scar size and improved NYHA classification for both groups; increased ejection fraction (1 × 10^8^ group) and proBNP (2 × 10^7^ group)	[162]
	Ischemic cardiomyopathy	Autologous or allogeneic BM-MSCs	2 × 10^7^, 1 × 10^8^, or 2 × 10^8^	Transendocardial injection in 10 left ventricular sites	Reduced scar size accompanied by decreased end-diastolic and end-systolic volume, increased ejection fraction; improved sphericity index; functional improvement in autologous group assessed by 6MWT	[127]
	Nonischemic dilated cardiomyopathy	Autologous or allogeneic BM-MSCs	1 × 10^8^	Transendocardial injection in 10 left ventricular sites	Increased ejection fraction (EF) and 6MWT in allogeneic BM-MCSc group compared to autologous group; allogeneic BM-MCSc group showed improved endothelial function, suppression of TNFα, functional capacity, and quality of life compared to allogeneic group; serious adverse events occurred in 28.2% of allogeneic group and 63.5% of autologous group	[126]
	Ischemic heart disease and ischemic heart failure (IHF)	Allogeneic ASCs	1.1 × 10^8^	Intramyocardial injection	Decrease left ventricular end-systolic volume, increased LVEF and exercise capacity	[167]
	Heart failure (HF) with reduced ejection fraction (HFrEF)	Allogeneic UC-MSCs	1 × 10^6^/kg	Intravenous	Increased LVEF, improved NYHA classification and MLHFQ	[165]
	Acute myocardial infarction (AMI)	Wharton’s jelly-derived MSCs	6 × 10^6^	Intracoronary infusion into the infarct artery	Improved cardiac function reflected by absolute increase in myocardial viability, perfusion within infarcted territory (SPECT); absolute increase in LVEF; absolute decreases in LV end-systolic and end-diastolic volume	[166]
Kidney	Renal fibrosis on peritoneal dialysis (PD)	Autologous ASCs	1.2 ± 0.1 × 10^6^/kg	Intravenous (cubital vein)	Decline in rate of solute transport across peritoneum determined by PET and D/Pcr	[66]
	Atherosclerotic renovascular disease (RVD)	Autologous ASCs	1 × 10^5^, 2.5 × 10^5^/kg	Intra-arterial infusion	Increased renal tissue oxygenation and cortical blood flow	[68]
Uterus	Asherman’s syndrome and endometrial atrophy	Autologous BM-MSCs	Mean 6.53 × 10^7^ (range: 1.9 × 10^7^ to 2 × 10^8^)	Transmyometrial implant in subendometrial zone	Improved uterine cavity, increased endometrial thickness; improved menstrual duration and intensity and pregnancy rates	[225]
	Asherman’s syndrome and endometrial atrophy	Autologous CD133^+^ BM-SCs	Mean 1.23 × 10^8^ (range: 4.2 × 10^7^ to 2 × 10^8^)	Intra-arterial catherization	Improved uterine cavity and increased endometrial thickness; increased mature vessel density, duration and intensity of menses, and pregnancy rates	[229]
	Asherman’s syndrome	Autologous SVFs	4.6 ± 0.7 × 10^6^	Intrauterine	Increased endometrial thickness and menstrual bleeding	[230]
	Recurrent intrauterine adhesions (IUAs)	Allogeneic UC-MSCs in collagen scaffold	1 × 10^7^	Infusion in uterine wall via catheter placed in uterine cavity	Increased endometrial thickness and expression levels of ERα, Ki67, and vWF; resumed menses and increased menstrual bleeding; improved uterine cavity and pregnancy rates	[231]
	Asherman’s syndrome	Autologous menstrual blood-derived stromal cells (menSCs)	1.0 × 10^6^	Intrauterine	Increased endometrial thickness and pregnancy rates	[232]
Skin	Systemic sclerosis	Autologous CD34^+^ HSCs	Mean dose 5.63 × 10^6^ /kg	Intravenous	Improved skin thickness assessed by mRSS	[251]
	Systemic sclerosis	Autologous CD34^+^ HSCs	Median dose 5.6 × 10^6^/kg	N/A (infusion)	Improved skin thickness based on mRSS	[254]
	Systemic sclerosis	Autologous CD34^+^ HSCs	≥2 × 10^6^/kg	N/A (infusion)	Improved skin thickness based on mRSS	[253]
	Systemic sclerosis	Autologous HSCs	N/A	N/A (infusion)	Improved skin thickness based on mRSS	[252]
	Systemic sclerosis	Autologous SVFs	Mean 3.76 ± 1.85 × 10^6^	Injection into subcutaneous tissue in contact with neurovascular pedicles	Reduced pain, finger circumference, and Raynaud’s severity; increased grip strength; decreased dystrophic capillaries and vascular suppression score	[256]
	Systemic sclerosis	Autologous ASCs	4 × 10^6^ to 8 × 10^6^	Injection into patient-specific area (face, arm, foot, limb)	Regression of dyschromia and reduced erythema; improved skin softening and sensitivity	[255]
	Systemic sclerosis	Autologous fat-enriched multipotent stem cells	N/A	Injection into oro-facial tissues	Improved mouth function and facial volumetric appearance	[257]
	Systemic sclerosis	Autologous fat-enriched multipotent stem cells	N/A	Local injection into oro-facial tissues	Increased interincisal distance and oral perimeter; improved neovascularization	[258]

Abbreviations: *αSMA*, α-smooth muscle actin; ASCs, adipose-derived stem cells; ALB, serum albumin; ALT, alanine aminotransferase; AST, aspartate transaminase; BM-MSCs, bone marrow mesenchymal stem cells; COL, collagen; D/P cr, dialysate-to-creatinine ratio; HGF, hepatocyte growth factor; HA, hyaluronic acid; HSCs, hematopoietic stem cells; IL, interleukin; INR, international normalized ratio; DLCO, diffusing capacity of the lung for carbon monoxide; EF, ejection fraction; ERα, estrogen receptor alpha; FVC, forced ventilation capacity; LVEF, left ventricular ejection fraction; N/A, not available; MELD, model for end-stage liver disease; MIF, macrophage migration inhibitory factor; MLHFQ, Minnesota Living with Heart Failure Questionnaire; NYHA, New York Heart Association class; mRSS, modified Rodnan’s skin score; PET, peritoneal equilibration test; proBNP, pro-brain natriuretic peptide; PT, prothrombin time; SPECT, single-photon emission computed tomography; SVFs, stromal vascular fraction cells; TBIL, total bilirubin; TGFβ1, transforming growth factor-1; TNFα, tumor necrosis factor alpha; 6MWT, six minute walk test; UC-MSCs, umbilical cord mesenchymal stem cells; vWF, von Willebrand factor.

## 4. Limitations

The clinical application of stem cells, especially human ESCs (hESCs), iPSCs, and iPSC-derived cells, raises several ethical and safety concerns. Ethical issues concerning stem cells are mainly related to the source of hESCs and iPSCs, while safety issues are related to the potential of all stem cells to transform into undesirable cell types. The main ethical dilemma concerning hESCs is their origin and the destruction of human embryos to obtain them. Other controversies are related to their immunogenic potential and risk of cancer formation. The plasticity of hESCs allows them to create several cell types; however, this ability makes them challenging to control after in vivo transplantation [259]. Once undifferentiated hESCs are transplanted, there is a huge risk that teratomas and tumors containing all three germ layers will develop [260]. It is estimated that teratomas develop in 33–100% of hESC-transplanted immunodeficient mice [261,262]. To be sure that teratomas will not develop, hESCs should be differentiated into desired and mature cell types before transplantation and then monitored for the presence of undifferentiated cells [263].

Despite the similarities between iPSs and hESCs in terms of karyotype, phenotype, telomerase activity, and differentiation capacity, they are ethically superior to hESCs, as their generation does not involve the destruction of embryos [264]. The main safety issue regarding the transplantation of iPSCs and iPSC-derived cells is their undesired differentiation and malignant transformation. Moreover, the genomic instability of iPSCs, along with uncontrolled proliferation and differentiation, may lead to the formation of tumors and/or undesired differentiation into a variety of somatic cells [54]. Thus, effective methods for the generation of purified populations of autologous iPSC-derived differentiated cells still remain a challenge for personalized and regenerative medicine [265].

Along with the promising results of MSC-based therapy, safety issues regarding treatment with MSCs are still a matter of debate. Similar to the aforementioned ESCs, iPSC, and iPSC-derived cells, MSCs also have the capacity to differentiate into undesired tissue, such as bone and cartilage [266,267]. Probably the local microenvironment in the tissues of recipients contains factors that promote unwanted differentiation of transplanted MSCs. In addition, MSCs promote metastasis by inducing angiogenesis, as they have the potential to differentiate into endothelial cells and create capillary tubular networks [268,269].

A very important issue in the context of the treatment of fibrosis is myofibroblast differentiation from stem cells. Indeed, using genetic lineage tracing technology, it has been demonstrated that diverse organ-resident perivascular MSC-like cells and BM-MSCs are implicated in myofibroblast generation during fibrosis [270,271]. Moreover, there is experimental evidence demonstrating that BM-MSCc transplanted into mice with chemically induced cirrhosis differentiate into myofibroblasts and contribute to hepatic fibrosis rather than providing an antifibrotic therapeutic element [114]. Thus, follow-up studies should pay special attention to this aspect of stem cell properties [18]. Since the results of studies indicate that MSCs can differentiate into undesired cell types and can also transform into tumors [272], studies that use MSCs in fibrotic disorders should be more focused on monitoring and long-term follow-up of MSC recipients. Concerning clinical trials conducted using stem cells as a treatment for organ fibrosis, the results have generally been optimistic, as several early-phase clinical studies have demonstrated encouraging effects on organ function and an acceptable safety profile in relatively short-term follow-up. Nevertheless, for the best clinical outcome, there is still a need to optimize therapeutic regimens, including the cell source, dose, administration route and frequency, the timing of delivery, and long-term safety.

## 5. Conclusions

In recent years, the understanding of the pathophysiology of organ fibrosis has significantly progressed. However, efficient therapy to stop the progression of fibrotic diseases or reverse them does not exist. At present, antifibrotic therapies are aimed at stabilizing the fibrotic process, halting inflammation, relieving symptoms, and improving the quality of life of patients, but the long-term goal is to reverse these destructive diseases and develop novel strategies to cure them. Results obtained from completed and ongoing clinical studies demonstrate the promising therapeutic potential of stem cell-based therapy in the treatment of fibrotic diseases in the lungs, liver, kidney, heart, and even the uterus and skin. Moreover, a number of patent applications for stem cell-based therapies in organ fibrosis have been filed worldwide (see examples in Table 2). As we described in this review, stem cells obtained from different sources such as bone marrow, adipose tissue, the pluripotent inner cell mass of the pre-implantation embryo, placenta, umbilical cord, Wharton’s jelly, and even menstrual blood have been used by researchers and clinicians to develop antifibrotic therapy. As we show here, several preclinical and clinical studies on stem cell-based therapy have been carried out to date. Nevertheless, there is still no specific stem cell-based therapy or extra-clinical treatment available to the general public.

The results of a large body of studies indicate a strong impact of the application of stem cells on fibrotic markers in in vitro research and clinical trials. However, the exact mechanisms of action of stem cells remain poorly understood and demand further investigation. Ascertaining these mechanisms would help to improve the efficiency of stem cell-based therapies and the survival rate of people suffering from organ fibrosis. Furthermore, many issues still have to be addressed before stem cells can be used clinically for fibrotic diseases, including sufficient cell numbers, optimal time, and optimal delivery route for cell transplantation. The lack of standardized and optimized protocols for stem cell isolation and culture expansion must also be resolved to assure reproducibility in clinical settings. Additionally, taking into consideration the safety issues, the development of stem cell-based therapy for fibrotic disorders should be more focused on constant observation and long-term follow-up of treated animal models to define possible detrimental effects and increase the clinical safety and efficacy.

## Figures and Tables

**Table 2:** Examples of patent applications for stem cell-based therapies in organ fibrosis (5 October 2021; Espacenet Patent search; https://worldwide.espacenet.com).

No.	Patent Name	Patent Number	Applicant
1.	Mesenchymal stromal cells and extracellular vesicles for treating viral infections, inflammation, and tissue fibrosis	WO2021181399A1	Exostem Biotec Ltd. (Petah Tikva, Israel)
2.	Biomarker composition for predicting the therapeutic efficacy of mesenchymal stem cells in renal fibrosis	KR102242286B1	Corestem Co Ltd. (Seongnam, South Korea); Chungbunk National University (Cheongju, South Korea)
3.	Generation of quiescent cardiac fibroblasts from human induced pluripotent stem cells for in vitro modeling of cardiac fibrosis	WO2020264308A1	Leland Stanford Junior University (Stanford, CA, United States)
4.	Mesenchymal stem cells and application thereof to treatment of acute lung injury, acute respiratory distress or pulmonary fibrosis	CN111904980A	IPM Biopharm Co Ltd. (Hangzhou, China)
5.	Method for improving living quality of patient with liver cirrhosis by applying stem cell therapy	CN111135192A	CAR-T (Shanghai, China) Bitechnology Co Ltd. (Guangzhou, China)
6.	Mesenchymal stem cell anti-liver fibrosis injection	CN110755454A	Qingdao re Store Biotechnology Co Ltd. (Qingdao, China)
7.	Composition for preventing or treating of liver fibrosis comprising exosomes derived from adipose stem cells as an active ingredient	KR102159791B1	ExoStemTech (Ansan, South Korea)
8.	Treatment of pulmonary fibrosis by atomizing inhalation of stem cell active peptide and preparation of atomizing agent	CN110403958A	Chenxin Shanghai Medical Tech Co Ltd. (Shanghai, China)
9.	Use of umbilical mesenchymal stem cells for treating pulmonary fibrosis	TW201838636A	National Yang Ming University (Taipei, Taiwan); Fu Yu Show (Taipei, Taiwan)
10.	Application of endometrial stem cells in preparation of drugs for preventing or treating pulmonary fibrosis	CN108969539A	Southern Medical University (Guangzhou, China); Guangdong Shengsai Biological Tech Co Ltd. (Guangzhou, China)
11.	Mesenchymal stem cell line useful for developing fibrosis therapeutic agent	KR20180093396A	Osteoneurogen (Seoul, South Korea)
12.	Production method of enhanced hepatocyte regenerating mesenchymal stem cell and Composition for treating liver cirrhosis using the same	KR101903964B1	Catholic Kwandong University (Gangneung, South Korea)
13.	Human adipose-derived mesenchymal stem cell anti-hepatic fibrosis injection and preparation method thereof	CN107496456A	Qingdao re Store Biotechnology Co Ltd. (Qingdao, China)
14.	Treatment of fibrosis using deep tissue heating and stem cell therapy	US2017232276A1	Primegen Biotech Llc (Santa Ana, CA, USA)
15.	Stem cell preparation capable of resisting hepatic fibrosis and preparation method of stem cell preparation	CN106038596A	Shenzhen Istem Regenerative Medicine Sci-Tech Co Ltd. (Shenzhen, China)
16.	Composition for preventing and treating liver fibrosis or liver cirrhosis, containing, as active ingredient, mesenchymal stem cells derived from human embryonic stem cells	US2016151420A1	Seoul National University Hospital (Seoul, South Korea)
17.	Application of exosome derived from human mesenchymal stem cells to resistance to tissue fibrosis and scar forming	CN105477016A	PLA Second Military Medical University (Shanghai, China)
18.	Application of umbilical cord mesenchymal stem cells in preparation of pharmaceutical preparation for treating PF (pulmonary fibrosis)	CN104666347B	The First Affiliated Hospital of Guangzhou Medical University (Guangzhou, China); Shenzhen Beike Biotechnology Co Ltd. (Shenzhen, China)
19.	Stem cell preparation for treating hepatic fibrosis	CN104622902B	Hangzhou S-Evans Ketuo Stem Cell Technology Res Co Ltd. (Hangzhou, China)
20.	Application of gene modified mesenchymal stem cell in pulmonary fibrosis treatment	CN103203025A	INST Radiation Med AMMS PLA (Beijing, China)
21.	Human umbilical cord mesenchymal stem cell (HUMSC) anti-hepatic fibrosis injection and preparation method thereof	CN102008507B	Tianjin Heze Stem Cells Technology Co Ltd. (Tianjin, China)
22.	Pharmaceutical composition for preventing and treating liver fibrosis or hepatic cirrhosis comprising mesenchymal stem cell	EP3878432A1	Seoul National University Foundation (Seoul, South Korea) RNL Bio Co Ltd. (Seoul, South Korea)
23.	Use of stroma stem cell derived from bone marrow in preparing formulation for treating hepatic fibrosis	CN1803192A	Sun Yat-sen University (Guangzhou, China)
24.	Stem Cell Therapy in endometrial pathologies	AU2015275798B2	Igenomix S.L. (Paterna, Spain)
25.	Compositions and methods for ameliorating tissue injury, enhancing liver regeneration and stem cell therapies	WO2020005891A1	University of Southern California (Los Angeles, CA, USA)
26.	ROR-1-positive mesenchymal stem cell-containing pharmaceutical composition for preventing or treating disease associated with fibrosis, method for preparing same, and method for preventing or treating disease associated with fibrosis using ROR-1-positive mesenchymal stem cells	WO2018164228A1	Rohto Pharmaceutical Co Ltd. (Osaka, Japan)
27.	Composition containing adipose stem cell-derived exosomes as active ingredient for preventing or treating liver fibrosis	WO2018093233	ExoStemTech Co Ltd. (Ansan, South Korea)
28.	Pluripotent stem cells that induces repair and regeneration after myocardial infarction	EP3659612	Clio Inc. (Tokyo, Japan); Gifu University (Gifu, Japan); Tohoku University (Sendai, Japan)
29.	Prophylactic or therapeutic agent for organ fibrosis	WO2005082402A1	Tohoku University (Sendai, Japan); Life Science Institute, Inc. (Tokyo, Japan)
30.	Stem cells for treating lung diseases	US20090274665A1	Cell Therapy Technologies Inc. (Vancouver, BC, Canada); Thebaud Bernard (Canada)
31.	Tissue repair by activated cells	CA3111750A1	Technion Research and Development Foundation Limited (Haifa, Israel)
32.	Compositions and methods directed to treating liver fibrosis	US2021087218A1	Alnylam Pharmaceuticals Inc. (Cambridge, MA, USA)
33.	Methods of reversing liver fibrosis using stem cells therapy	CN111281885A	CAR-T (Shanghai) Biotechnology Co Ltd. (Shanghai, China)
34.	Composition for preventing or treating pulmonary fibrosis comprising exosomes extracted form adipose-derived stem cells	CN109562129A	Hanyang University ERICA Industry-University Cooperation Foundation (Hanyang, China)
35.	Mesenchymal stem cell line useful for development of fibrosis therapeutic agent	WO2018147504A1	Osteoneurogen (South Korea)
36.	Umbilical cord mesenchymal stem cells for treating lung diseases and preparation method thereof	CN111518758A	Shenzhen Hornectorn Biotechnology Co Ltd. (Shenzhen, China)
37.	Stem cell preparation for treating liver cirrhosis	CN106109497A	Shenzhen Istem Regenerative Medicine Sci-Tech Co Ltd. (Shenzhen, China)
38.	Use of umbilical cord mesenchymal stem cells for the treatment of pulmonary fibrosis	CN108324736A	Fu Yuxiu (China)
39.	Use of human fat-derived mesenchymal stem cells in treatment of diseases in kidney and ocular fundus	CN102048756A	Institute of Basic Medical Sciences, Chinese Academy of Medical Sciences (Beijing, China)
40.	Mesenchymal stem cells for use in improving pulmonary function	EP3653217A1	Mesoblast International Sárl (Switzerland)
41.	Uterine blood stem cells and exosomes for treating intrauterine adhesion	CN111979199A	Zhejiang Puhui Medical Technology Co Ltd. (Hangzhou, China)

## Abbreviations

6MWT	6-min walk test
αSMA	α-smooth muscle actin
ACTA2	α-smooth muscle actin
ADPKD	autosomal-dominant polycystic kidney disease
AECs	alveolar epithelial cells
AFSCs	amniotic fluid-derived stem cells
AKI	acute kidney injury
Akt	protein kinase B
ALB	serum albumin
ALT	alanine aminotransferase
AS	Asherman syndrome
AST	aspartate aminotransferase
ASCs	adipose-derived stem cells
ASCs-CM	conditioned medium from ASCs
AQP	aquaporin
BLM	bleomycin
bFGF	basic fibroblast growth factor
BMI	body mass index
BM-MSCs	bone marrow mesenchymal stem cells
BMPCs	bone marrow progenitor cells
CCl_4_	carbon tetrachloride
COL	collagen
CTGF	connective tissue growth factor
dcSS	diffuse cutaneous systemic sclerosis
DDIT3	DNA-damage inducible transcript 3
Dkk-1	Dickkopf-1
DLCO	carbon monoxide diffusing capacity
D/P cr	dialysate-to-creatinine ratio
EBs	embryoid bodies
ECM	extracellular matrix
EF	ejection fraction
eGFR	estimated glomerular filtration rate
Eln	elastin
eMSC	endometrial MSCs
EMT	epithelial-to-mesenchymal transition process
ERα	estrogen receptor alpha
ERK	extracellular-signal-regulated kinase
ESCs	embryonic stem cells
Ev	extracellular vesicles
FN	fibronectin
FVC	forced vital capacity
G-CSF	granulocyte colony-stimulating factor
GDNF	glial cell line-derived neurotrophic factor
GFP	green fluorescent protein
GM-CSF	granulocyte-macrophage colony-stimulating factor
GPSCs	germline cell-derived pluripotent stem cells
GTCs	tubular-like cells
GVHD	graft-versus-host disease
HA	hyaluronic acid
hAMSCs	human amniotic mesenchymal stromal cells
HBO	hyperbaric oxygen
HBV	hepatitis B
HCV	hepatitis C
HGF	hepatocyte growth factor
HLA	histocompatibility complex
HOCl	hypochlorous acid
HSCs	hematopoietic stem cells
IGF-1	insulin growth factor 1
IL4Rα	IL-4 receptor alpha
IL	interleukin
ILCs	insulin-producing islet-like clusters
INR	international normalized ratio
IPF	idiopathic pulmonary fibrosis
iPSCs	induced pluripotent stem cells
IUAs	intrauterine adhesions
JNK	JUN N-terminal kinase
lcSS	limited cutaneous systemic sclerosis
LVEF	left ventricular ejection fraction
MCP-1	monocyte chemotactic protein-1
MELD	model for end-stage liver disease
MI	myocardial infarction
MIF	macrophage migration inhibitory factor
MLHFQ	Minnesota living with heart failure questionnaire
MMPs	matrix metalloproteinases
mRSS	modified Rodnan’s skin score
MSCs	mesenchymal stem cells
MT1-MMP	membrane-bound matrix metalloproteinases
NAFLD	non-alcoholic fatty liver disease
NASH	non-alcoholic steatohepatitis
NYHA	New York Heart Association class
PAI-1	plasminogen activator inhibitor 1
PCNA	proliferating cell nuclear antigen
PDE	phosphodiesterase
PET	peritoneal equilibration test
PF	pulmonary fibrosis
proBNP	pro-brain natriuretic peptide
PT	prothrombin time
PVD	portal vein diameter
ROCK	RHO-associated kinase
RPCs	renal progenitor cells
RVD	renovascular disease
SDF-1α	stromal cell-derived factor 1 alpha
SHH	sonic hedgehog
SPC	surfactant protein C
SPECT	single-photon emission computed tomography
SS	systemic sclerosis
SVF	stromal vascular fraction
TBIL	total bilirubin
TECs	tubular epithelial cells
TGF- β	transforming growth factor β
TGFβRI	TGFβ receptor type 1
Thbs1	thrombospondin 1
TIMPs	tissue inhibitors of matrix metalloproteinases
TNF	tumor necrosis factor
TTA	thioacetamide
UC-MSC	umbilical cord MSCs
vWF	von Willebrand factor
